# Genetically modifying skin microbe to produce violacein and augmenting microbiome did not defend Panamanian golden frogs from disease

**DOI:** 10.1038/s43705-021-00044-w

**Published:** 2021-10-18

**Authors:** Matthew H. Becker, Jennifer A. N. Brophy, Kevin Barrett, Ed Bronikowski, Matthew Evans, Emerson Glassey, Alyssa W. Kaganer, Blake Klocke, Elliot Lassiter, Adam J. Meyer, Carly R. Muletz-Wolz, Robert C. Fleischer, Christopher A. Voigt, Brian Gratwicke

**Affiliations:** 1 Smithsonian’s National Zoo and Conservation Biology Institute, Center for Species Survival, Front Royal, VA USA; 2 Synthetic Biology Center, Department of Biological Engineering, Massachusetts Institute of Technology, Cambridge, MA USA; 3 Maryland Zoo in Baltimore, Baltimore, MD USA; 4 Smithsonian’s National Zoo and Conservation Biology Institute Reptile Discovery Center, Washington, DC USA; 5 Department of Environmental Science and Policy, George Mason University, Fairfax, VA USA; 6 Smithsonian’s National Zoo and Conservation Biology Institute, Center for Conservation Genetics, Washington, DC 20001 USA; 7 Present Address: Liberty University Department of Biology and Chemistry, Lynchburg, VA USA

**Keywords:** Microbiome, Bacterial genetics, Applied microbiology

## Abstract

We designed two probiotic treatments to control chytridiomycosis caused by *Batrachochytrium dendrobatidis* (Bd) on infected Panamanian golden frogs (*Atelopus zeteki*), a species that is thought to be extinct in the wild due to Bd. The first approach disrupted the existing skin microbe community with antibiotics then exposed the frogs to a core golden frog skin microbe (*Diaphorobacter* sp.) that we genetically modified to produce high titers of violacein, a known antifungal compound. One day following probiotic treatment, the engineered *Diaphorobacter* and the violacein-producing pathway could be detected on the frogs but the treatment failed to improve frog survival when exposed to Bd. The second approach exposed frogs to the genetically modified bacterium mixed into a consortium with six other known anti-Bd bacteria isolated from captive *A. zeteki*, with no preliminary antibiotic treatment. The consortium treatment increased the frequency and abundance of three probiotic isolates (*Janthinobacterium*, *Chryseobacterium*, and *Stenotrophomonas*) and these persisted on the skin 4 weeks after probiotic treatment. There was a temporary increase in the frequency and abundance of three other probiotics isolates (*Masillia, Serratia*, and *Pseudomonas*) and the engineered *Diaphorobacter* isolate, but they subsequently disappeared from the skin. This treatment also failed to reduce frog mortality upon exposure.

## Introduction

Chytridiomycosis, a disease caused by the fungal pathogen *Batrachochytrium dendrobatidis* (Bd), is responsible for the presumed extinction of 90 amphibian species and has devastated amphibian populations around the globe, particularly in the mountainous neotropics [1]. Harlequin toads of the genus *Atelopus* have been among the taxa most impacted by this disease [2,3], and the Panamanian golden frog *Atelopus zeteki* is thought to be extinct in the wild as a result of chytridiomycosis-related declines, but large ex-situ populations have prevented their extinction [3].

Despite one unique example of eliminating Bd in nature [4], there is a dearth of effective solutions available to control the amphibian chytrid fungus in wild situations [5]. In other disease systems, probiotic therapies have controlled diseases through different mechanisms including (i) modulating host innate immunity or inflammatory responses [6,7]; (ii) reducing the ability of a vector host to transmit the disease [8]; (iii) restoring disrupted microbiomes that usually have a protective function [9]; (iv) directly inhibiting pathogen growth [10–12]; or (v) neutralizing pathogen toxins [13]. Genetic engineering tools have allowed further development of designer probiotic therapies that could have enormous potential applications for managing disease [14,15]. Examples include symbionts that have been genetically engineered to express antigens that sense and kill pathogenic bacteria [14], suppress viral infections and reduce mite survival in bee colonies [16] and neutralize cholera toxins [17].

Panamanian golden frogs skin biota are diverse, with more than 3000 operational taxonomic units (OTUs) detected on captive frogs, that share 70% of their skin flora with wild frogs [18]. The skin flora is dominated by 15 core OTUs in the phyla ﻿Bacteroidetes, Actinobacteria, and Proteobacteria that are found on more than 90% of captive and wild frogs [18]. One of the core OTUs is a rod-shaped denitrifying bacteria in the genus *Diaphorobacter*, a genus that has also been found in sludge, air, and rice paddies [19–21]. Probiotics therapies with antifungal bacteria have been proposed as a way to directly inhibit Bd growth [22,23]. There is evidence that this approach can help to prevent or reduce Bd infections in vitro and in vivo through the production of potent antifungal metabolites including violacein produced by *Janthinobacterium* and prodigiosin from *Serratia* [12,24–27]. When probiotic *Serratia* bacteria were genetically modified in a knockout experiment to impair the production of prodigioson, frogs fared worse when exposed to Bd [28]. Despite these examples, previous probiotic supplementation of Panamanian golden frog skin with antifungal microbes did not mitigate the disease [29,30]. In these previous experiments, probiotics sourced from other amphibian species were applied individually to the frogs and they colonized the skin poorly, or did not achieve sufficient densities to have a protective function, leading to a re-examination of our probiotic approach.

We tested two novel probiotic strategies to reduce Bd infections in Panamanian golden frogs. First, we sampled the microbiota on the skin of captive Panamanian golden frogs, characterized the microbiome using 16S rRNA sequencing, and attempted to isolate the most prevalent and abundant strains of bacteria defined as core microbes [18]. These were selected as candidates for genetic manipulation to include the violacein pathway, which is well-characterized and reproducible [12,25,27,31]. By using a core skin microbe that is known to thrive on golden frog skin [18], we hoped to avoid a negative immune response from the frog to the probiotic treatment [32], and achieve sufficient threshold densities to obtain a probiotic effect [12,29]. Second, we aimed to increase the likelihood that probiotics would persist on the frog skin using a multi-genus consortium approach postulated to be more effective at mitigating disease than single-species probiotic treatments [33,34]. We mixed a ‘cocktail’ of several different anti-Bd bacteria isolated from Panamanian golden frogs, and even though some were similar to previous probiotic isolates attempted [12,29], we expected that sourcing the probiotic bacteria from golden frogs would result in strains that are better adapted to golden frog skin.

## Materials and methods

### Golden frog animal handling and skin microbiome experiments

We received endangered species permits from the USFWS (FWS/DMA/PRT-067210) and IACUC approvals from the Maryland Zoo in Baltimore, and the National Zoological Park (#14-35). We subsampled 200 animals from all existing genetic lines of surplus-bred Panamanian golden frogs held at the Maryland Zoo in Baltimore. We swabbed each animal twice, ten times on the belly, ten times on each thigh, and 5 times on each foot [35]. DNA from the first swab was extracted for 16S amplicon sequencing [18] (details below) to identify species based on their 16S gene similarity to sequences deposited in the Ribosomal Database Project (RDP) [36], and relative abundance of bacteria on the frog skin used to identify core skin microbes. We chose a subset (*n* = 18) of these individuals that carried potential anti-Bd bacteria identified in a global database [37], and the second swab was cryopreserved in a glycerol solution from these 18 animals to culture and isolate the bacteria on R2A media plates [38].

### Measurement of Bd inhibition by skin isolates

To determine which golden frog bacterial isolates secreted anti-Bd metabolites, we used an in vitro challenge assay in a 96-well plate format using spectrophotometry [30] with Bd strain JEL 423 and selected cultured isolates from the 18 golden frogs mentioned above. These bacteria were chosen based on relatedness to core golden frog microbes or to known putative anti-Bd isolates [37] based on 16S rRNA gene sequence similarity.

### Screen for antibiotic resistance of skin isolates

Single colonies were inoculated into 2 mL of Luria Broth (LB) in 15 mL culture tubes (Fischer Scientific 352059) and incubated overnight at 30 °C and 250 rpm in a New Brunswick Scientific Innova 44. In the morning, starter cultures were diluted 1:200 in 2 mL of fresh LB medium and incubated for 4 h at the same temperature and speed. After 4 h, cultures were serially diluted and plated onto LB agar plates containing antibiotics. Strains were deemed resistant to an antibiotic when the ten-fold dilution resulted in colonies.

### Cloning strains, media and chemicals

*Escherichia coli* strain NEB10β (Δ(ara-leu) 7697 araD139 fhuA ΔlacX74 galK16 galE15 e14- ϕ80dlacZΔM15 recA1 relA1 endA1 nupG rpsL (Str^R^) rph spoT1 Δ(mrr-hsdRMS-mcrBC)), *E. coli* strain JTK164D (F’ Δ(ara-leu)7697 [araD139]B/r Δ(codB-lacI)3 galK16 galE15 λ- e14- mcrA0 relA1 rpsL150(StrR) spoT1 mcrB1 hsdR2(r-m+) purM(pir TpR)), were used to construct all plasmids. *E. coli* strain HB101 (F- mcrB mrr hsdS20(rB- mB-) recA13 leuB6 ara-14 proA2 lacY1 galK2 xyl-5 mtl-1 rpsL20(Sm^R^) glnV44 λ-) containing helper plasmid pRK600 (Cm^R^) was used to perform conjugations. *E. coli* strains were grown at 37 °C in LB medium (Becton Dickinson 244630). Strains isolated from frog skin were grown at 30 °C in LB or Reasoner’s 2A (R2A) medium (Teknova R0005). Antibiotics and other chemicals were used at the following concentrations: carbenicillin (100 μg/mL) (Gold Bio C-103), kanamycin (50 μg/mL) (Gold Bio K-120), tetracycline (10 μg/mL) (Gold Bio T-101), spectinomycin (100 μg/mL) (Gold Bio S-140), chloramphenicol (35 μg/mL) (USB Corporation 23660), isopropyl β−D-1-thiogalactopyranoside (IPTG) (1 mM) (Gold Bio I2481C).

### Conjugation experiments

Conjugation was selected as the method of introducing DNA to the bacteria because it is a relatively simple procedure that works for a wide range of species [39]. Plasmids were introduced to the golden frog skin microbe *Diaphorobacter* 63F by conjugation using the triparental mating method. Briefly, single colonies of the *E. coli* donor strain, *E. coli* helper strain, and *Diaphorobacter* 63F recipients were inoculated into 2 mL of LB medium with the appropriate antibiotics and grown overnight at 30 °C and 250 rpm. In the morning, cells were concentrated by centrifuging overnight cultures and resuspending in a ten-fold smaller volume of fresh LB medium. All three strains were mixed at an equal ratio (10 µL per strain) and spotted onto LB plates. Conjugations were incubated for 12 h at 30 °C. After the mating step, bacteria were scraped from the plates, resuspended in 1 mL LB and plated onto selective media. *E. coli* donor strain NEB10β was used for the conjugation of all BHR-containing plasmids and *pir* *+* donor strain JTK164D was used to conjugate the R6K plasmids for chromosomal integration.

### Violacein pathway engineering

Violacein pathway genes from *Chromobacterium violaceum* ATCC 12472 were amplified from iGEM registry plasmids BBa_K274002 (vioABCE) (http://parts.igem.org/Part:BBa_K274002) and BBa_K598020 http://parts.igem.org/Part:BBa_K598020) (vioD). To construct the *vioABDCE* RBS library, oligonucleotides with ten degenerate bases upstream of the start codon (NNNTGNNNNNNRTG) were used to amplify each gene in the pathway. Golden gate reactions were used to combine the pieces together. The resulting library was transformed into *E. coli* and conjugated into the *Diaphorobacter* 63F isolate using the triparental mating method described above. The sequence of the pathway selected is provided in Supplementary Table 1. Violacein measurements and validation methods are detailed in Supplementary methods.

### Fluorescence measurements

Single colonies were inoculated into 2 mL of LB medium containing kanamycin in 15 mL culture tubes (Fischer Scientific 352059) and incubated overnight at 30 °C and 250 rpm. In the morning, starter cultures were diluted 1:200 in 2 mL of fresh media with or without IPTG and grown for 4 h at the same temperature and speed. After 4 h, cultures were diluted 1:15 into phosphate-buffered saline and measured using a BD Biosciences Fortessa flow cytometer with a blue (488 nm) laser. An injection volume of 10 µL and flow rate of 0.5 µL/s were used. Cytometry data were analyzed using FlowJo (TreeStar Inc., Ashland, OR) and populations were gated on forward and side scatter. The gated populations consisted of at least 30,000 cells. The median fluorescence of the gated populations was calculated using FlowJo.

### Doubling time measurement

All strains were grown in incubated shakers at 250 rpm. Single colonies were inoculated into 2 mL of LB or R2A media in 15 mL culture tubes and incubated overnight at 30 °C and 250 rpm. In the morning, starter cultures were diluted 1:500 in 150 µL of fresh medium in black 96-well optical bottom plates (Thermo Scientific 165305) and incubated at 30 °C with 1 mm orbital shaking in an Biotek Synergy H1 plate reader. Optical density measurements at 600 nm wavelength were made every 20 m for 12 h. Doubling times were calculated using the slope of the linear portion of the growth curves during the exponential growth phase.

### Treatment of golden frogs with probiotics

We used 66 surplus-bred Panamanian golden frogs *Atelopus zeteki* provided by the Maryland Zoo in Baltimore, divided into the following treatment groups: Uninfected control (*n* = 10); Bd (*n* = 28); Bd + 63 F:vio (*n* = 12); and Bd + consortium (*n* = 17). Each frog was housed in an isolated cage (Supplementary Methods). For the Bd + 63F:vio group we applied antibiotics for 3 days prior to probiotic treatment using 100 mL of a solution containing 12 mg/L cephalexin, 14.5 mg/L sulfamethoxazole, and 2.9 mg/L trimethoprim. The purpose of this treatment was to disrupt the pre-existing core *Diaphorobacter* community prior to exposure to the synthetic strain. The day following the last antibiotic treatment, 100 mL of a solution containing the genetically modified *Diaphorobacter* 63F:vio was administered every other day for 10 days using a dose of 375,000 cells/mL for the first application, 750,000 cells/mL for the second, and 1.5 million cells/mL for the third, fourth, and fifth applications. The gradual increase of dose was conducted to reduce the likelihood of an immune shock reaction to the probiotics [32]. For the Bd + consortium group, no antibiotics were administered to the frogs, and 100 mL of a solution containing a total of 375,000 cells/mL of each probiotic listed in Supplementary Table 2 plus the *Diaphorobacter* 63F:vio strain were administered every other day for 10 days, employing the strategy of a consistent low dose per strain with an overall high total bacterial dose. We administered sham 100 mL treatments to control and Bd animals by pouring 98 mL of sterilized reverse-osmosis (RO) water into sterilized cages, soaking several autoclaved sheets of brown paper towel in the process, and spraying 2 mL of RO water directly on to the frog (Supplementary Methods). We administered all antibiotic and probiotic treatments using the same method, but with RO water containing antibiotics or probiotics at the concentrations listed above.

### Exposure of golden frogs to Bd

On day 0 of the challenge experiment (6/15/2016) individuals were placed in whirlpacks and exposed for 6 h to 20 mL of a solution containing 12,000 zoospores of Bd global pandemic lineage JEL 423 isolated from *Hylomantis lemur* in Panama. After the exposure period, the golden frogs were returned to their cages with the remaining Bd solution. During probiotic and Bd treatments, control individuals were treated exactly the same, but with RO water containing no added microorganisms. Daily husbandry of the golden frogs is provided in Supplementary Methods. The date of death of each frog was recorded in daily husbandry logs until the last day of the experiment (9/13/16, Day 90) when the experiment was censored and all surviving golden frogs were humanely euthanized by immersion in a buffered, saturated solution of 10 g/L MS222 [40].

### Sample collection and processing

All living animals were swabbed on day 0 (1 day after probiotic treatment and 1 day before Bd exposure—week 0), on day 29 (week 4), and on day 57 (week 8). ﻿We extracted DNA from each swab with a Qiagen DNeasy blood and tissue kit (Valencia, CA, USA) following the manufacturer’s protocol. ﻿We amplified extracted DNA for Bd analysis using qPCR methods [41], and quantified zoospore equivalents using standards prepared from Bd strain JEL 423. To assess cutaneous bacterial diversity via culture-independent methods, we prepared extracted DNA for 16S rRNA amplicon sequencing for weeks 0 and 4 [42], following methods previously described [18]. Briefly, DNA extracted from a sterile swab was included as a negative control in each set of extractions. We amplified prokaryotic DNA with primers that target the V4 region of the 16S rRNA gene, pooled amplicons in equimolar ratios, size-selected the library by gel extraction with Qiagen QiaQuick Gel Extraction Kit, and purified the final library with the Qiagen QIAquick PCR Purification Kit (Qiagen, Valencia, CA, USA) following the manufacturer’s protocol. We sequenced all samples and negative controls on an Illumina MiSeq instrument with 2 ×250 bp paired-end sequencing [42]. Bioinformatics and statistical analyses are detailed in Supplementary methods.

## Results

### Culturing skin microbes from Panamanian golden frogs

**Fig. 1:**
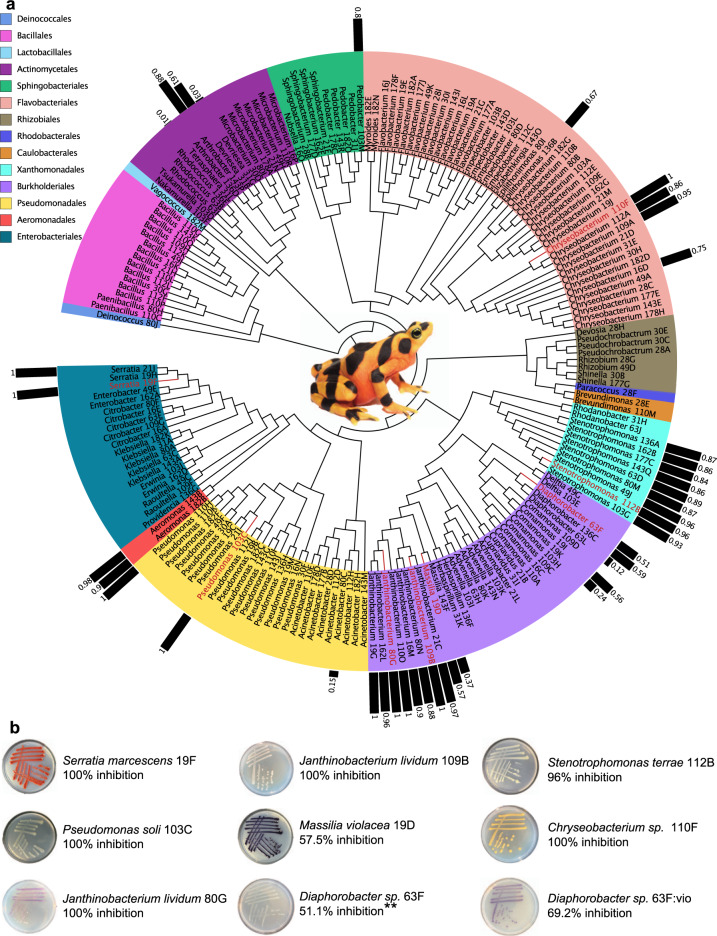
Culture-dependent isolate analysis of captive Panamanian golden frogs. **a** Phylogenetic tree of bacteria cultured from the skin of 18 golden frogs screened for the presence of known anti-Bd bacteria. Bars represent strength of anti-Bd inhibition for a subsample of candidate probiotics identified from an existing database, isolates used in the final probiotic experiment are highlighted in red. **b** Inhibitory isolates selected for the probiotic consortium, note the genetically modified *Diaphorobacter* 63F:vio was included in the probiotic cocktail treatment. **The core skin microbe *Diaphorobacter* 63F wild-type strain was not used in probiotic consortium, but is shown here to illustrate the colony color prior to genetic modification.

We subsampled 200 animals from all existing genetic lines of surplus-bred Panamanian golden frogs held at the Maryland Zoo in Baltimore. The DNA from one swab was extracted and the 16S rRNA gene was amplified using PCR in order to provide a culture-independent evaluation of the most abundant bacteria on the frog skin [18]. The second swab was retained in a growth media containing glycerol [43] and then cryopreserved for culturing. We chose a subset (*n* = 18) of these frogs that carried putative/predicted anti-Bd bacteria by comparing their OTUs to a global database of known anti-Bd microbes [37], and then cultured live bacteria from the second cryopreserved swab [38]. We identified isolates by amplifying and sequencing the 16S rRNA gene, and comparing it with 16S rRNA genes deposited in the RDP [18,36]. In total, we were able to culture 193 bacterial isolates from 18 golden frogs (average = 10.7 isolates per individual) (Fig. 1a). A majority of these isolates were identified as Gammaproteobacteria (31%), Flavobacteria (24%), and Betaproteobacteria (18%). We also compared the culture-independent 16S sequences collected from the 200 golden frogs with the cultured isolates’ 16S sequences to identify whether or not we were able to culture any core bacteria that dominated the golden frog skin bacterial community (average relative abundance ≥ 1% and prevalence ≥ 90% on all golden frogs). We were able to culture 13 out of 22 core bacteria (≥98% 16S rRNA gene sequence similarity); eight of these isolates served as candidates for genetic engineering (Table 1).

**Table 1:** Core Skin Bacteria cultured from *Atelopus zeteki* skin that were screened for potential to genetically modify them to include the violacein pathway.

Culture-independent OTU ID^a^	Relative abundance (%)^b^	Prevalence (%)^c^	Sequence similarity^d^	Culture-dependent isolate ID	RDP taxonomic identification (confidence estimate)
564487	10.3	100	100	103M	*Pedobacter sp*. (100%)
341094	3.2	97	98	143P	*Glutamicibacter sp*. (100%)
219549	2.8	99	100	16D	*Chryseobacterium sp*. (100%)
885496	2.5	99	100	31L	*Knoellia sp*. (56%)
denovo3687	2.5	99	100	31G	*Devriesea sp*. (62%)
253429	2.3	91	100	136B	*Chryseobacterium sp*. (100%)
1140286	1.1	99	98	63F	*Diaphorobacter sp*. (97%)
4449458	0.9	98	100	80C	*Acinetobacter sp*. (100%)

^a^OTU identification number from culture-independent analysis of 200 captive *Atelopus zeteki*

^b^Average percent relative abundance among all golden frogs from culture-independent analysis (*n* = 200)

^c^Prevalence among all golden frogs from culture-independent analysis (*n* = 200)

^d^Percent sequence similarity of the overlapping region (length = 253 bp) of 16S rRNA gene between the culture-independent OTU consensus sequence and the culture-dependent isolate sequence

An *in vitro* challenge assay was performed with Bd strain JEL 423 and selected golden frog bacterial isolates to determine which isolates secreted anti-Bd metabolites (Fig. 1a) [30]. We selected the most inhibitory isolate from each of six different genera (that do not contain known amphibian pathogens) for a probiotic cocktail application to golden frogs prior to Bd exposure (Fig. 1b and Supplementary Table 2). For one of these genera, *Janthinobacterium lividum* we selected two different morphotypes (white and purple) based on pre-existing interest in its probiotic potential [26,29] (Fig. 1, Supplementary Table 2).

### Engineering commensal bacteria to produce violacein

**Fig. 2:**
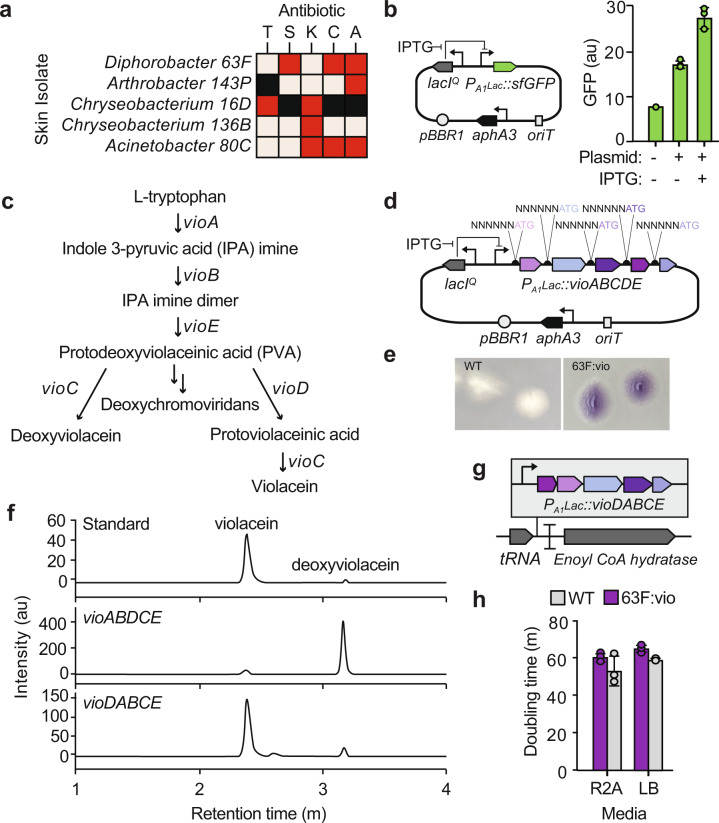
Engineering violacein production into Panamanian golden frog bacterial isolates. **a** Skin isolates were tested for resistance to carbenicilin (A, 100 μg/mL), chloramphenicol (C, 35 μg/mL), kanamycin (50 μg/mL), spectinomycin (S, 100 μg/mL), and tetracycline (T, 10 μg/mL). Strains were categorized as either resistant (red) or susceptible (pink) to the antibiotic (“Materials and methods”). Combinations that were not tested are colored black. **b** Fluorescence of *Diaphorobacter* 63F strains with (+) or without (−) plasmid pJAB582 (BHR lacI^Q^ P_A1_lacO-1-sfGFP oriT aphA3). IPTG (1 mM) was added to induce expression of *gfp* from pJAB582. The bar graph shows the average of three replicates, dots represent individual data points, and error bars are the s.d. **c** Violacein biosynthetic pathway. **d** Plasmid containing violacein pathway library. Ribosome binding sites were randomized using degenerate primers (“Materials and methods”). **e** Image of 63F strains grown on R2A media for 72 h at 23 °C. **f** HPLC-UV profiles of a violacein standard (top), 63F strain carrying plasmid pJAB631 (BHR P_A1_lacO-1-vioABDCE oriT aphA3) (middle), and engineered *Diaphorobacter* 63F:vio strain containing *P*_*A1*_*lacO-1-vioDABCE* integrated into the genome (bottom). The identity of the peaks was determined by LC-MS. **g** Placement of the violacein pathway in the *Diaphorobacter* 63F chromosome. **h** Doubling times of wild type (gray) and engineered (purple) 63F strains. The bar graph shows the average of three replicates, dots represent individual data points, and error bars are the s.d.

We evaluated eight of the most abundant bacterial strains on golden frog skin that grew in culture for their potential to be genetically modified (Supplementary Table 2). We first measured the strains’ resistance to several antibiotics that could be used for selection (tetracycline, spectinomycin, ampicillin, kanamycin). Strains that grew quickly in vitro (formed colonies after 48 h of growth at 30 °C) and were sensitive to at least one antibiotic were selected as candidates to introduce genes for violacein production (Fig. 2a).

First, we attempted to deliver a broad host range plasmid (BHR origin of replication from broad host range plasmid pBBR1 [44] containing an isopropyl-β-D-1 thiogalactopyranoside (IPTG)-inducible green fluorescent protein (GFP) reporter into the frog skin strains, sequence of the plasmid provided in Supplementary Table 3. This plasmid confers resistance to kanamycin, therefore we first attempted to engineer the kanamycin-sensitive strains *Diaphorobacter* 63F and *Glutamicibacter* 143P. Conjugation was selected as the method of introducing DNA to the bacteria because it is a relatively simple procedure that works for a wide range of species (“Materials and methods”) [39]. Only the *Diaphorobacter* isolate yielded kanamycin-resistant colonies after conjugation. Successful plasmid delivery was confirmed by PCR and GFP expression, as measured using flow cytometry (Fig. 2b). The addition of 1 mM IPTG to cells grown in culture yielded a modest increase in fluorescence after 4 h in culture, indicating that the promoter worked and could be used to express recombinant genes in this species.

A library of violacein pathways was generated to produce the antifungal metabolite in *Diaphorobacter* 63F. Five enzymes (VioABCDE) from the violacein-producing bacterium *Chromobacterium violaceum* are needed to convert l-tryptophan into violacein (Fig. 2c) [31]. To express *vioABDCE* in *Diaphorobacter*, we made a large ribosome binding site (RBS) library by randomizing ten of the nucleotides upstream of each gene in the pathway (Fig. 2d). Pathway variants were cloned into a BHR ori plasmid and conjugated from *E. coli* to *Diaphorobacter* 63F through a triparental mating and then plated and screened by eye. This method yielded two visibly purple colonies and violacein production was verified using LC-MS and both purple *Diaphorobacter* 63F colonies were producing deoxyviolacein, a byproduct of the violacein biosynthetic pathway (Fig. 2f).

Genes in the biosynthetic pathway were then reordered to optimize violacein production. The undesired compound deoxyviolacein is produced when violacein precursor protodeoxyviolaceinic acid is hydroxylated by VioC instead of oxidized by VioD (Fig. 2c). Thus, we sought to increase *vioD* expression in order to reduce the generation of deoxyviolacein and produce violacein. To increase the expression of VioD, we moved the *vioD* gene to the start of our synthetic violacein operon. In bacteria, the first gene in bacterial operons is often the most highly expressed and expression decreases as a function of the distance from the transcription start site [45]. Therefore, moving *vioD* to the beginning of the operon should increase its relative expression level. Strains with the *vioDABCE* operon produced high levels of violacein and deoxyviolacein was no longer detected (data not shown).

The pathway was integrated into the *Diaphorobacter* 63F genome to prepare the strain for transfer back to the golden frogs. Antibiotics are typically required for the maintenance of plasmids. Indeed, the violacein-containing plasmid was lost from *Diaphorobacter* 63F in the absence of kanamycin selection (data not shown). Since antibiotics would not be applied directly to the golden frogs after disease exposure, the violacein pathway was moved to the *Diaphorobacter* 63F chromosome to increase the stability of the engineered trait. The operon was integrated at the end of a tRNA operon, between a tRNA-met(CAT) gene and its terminator (Fig. 2g). This location was selected to minimize the impact of the new DNA on host fitness. tRNAs are a common location for mobile genetic elements [46] and do not encode enzymes, such as sugar hydrolases [47] or amino acid synthases [48], that may be essential for survival in vivo. The tRNA operon was identified by ARAGORN (a tRNA/tmRNA detection software) [49]. A hypothetical enoyl coA hydratase was identified just downstream of the tRNA operon using BLAST. We constructed a suicide vector with the R6K origin of replication and regions of homology to both the tRNA and hydratase genes to integrate the violacein pathway into this location. The R6K origin requires a transfactor (pi protein) to replicate [50]. When conjugated into the *Diaphorobacter* 63F strain, which does not contain pi, the plasmid was forced to integrate. We screened kanamycin-resistant colonies for the presence of double-crossovers of the violacein pathway into the correct location and found several with the pathway integrated into the correct location. Production of violacein in the strains with the integrated pathway was verified by LC-MS (Fig. 2f). The resulting violacein-producing *Diaphorobacter* 63F strain was named *Diaphorobacter* 63F:vio.

When considering the reintroduction of the engineered commensal strain to the skin (*Diaphorobacter* 63F:vio), it is important that the violacein pathway not impart a fitness burden. To this end, we tested the fitness of the engineered strain, relative to the unmodified *Diaphorobacter* 63F strain by co-culturing the strains in non-selective media. In both rich and minimal media, we did not observe a difference in growth between the wild-type and engineered strain in vitro (Fig. 2h). The sequence of the plasmid to construct *Diaphorobacter* 63F:vio is located in Supplementary Table 1.

### Probiotic treatment of Panamanian golden frogs

**Fig. 3:**
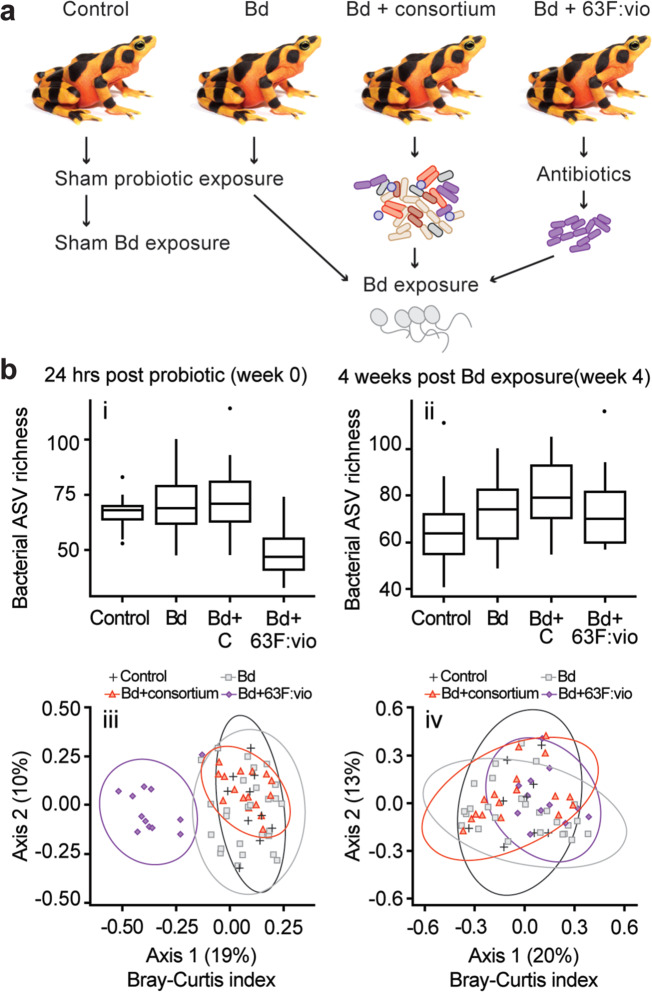
Bacterial community dynamics due to experimental treatment. **a** Treatments applied to Panamanian golden frogs. **b** Bacterial community response to experimental treatments, week 0 samples were taken 24 h after probiotic treatment and just prior to Bd exposure, and 4 week samples were taken 4 weeks after Bd exposure. Bacterial amplicon sequence variant (ASV) richness at week 0 was significantly different (ASV richness ANOVA, *F*_2,62_ = 14.22, *p* < 0.001), but not at week 4 (ASV richness ANOVA *p* = 0.24). Bacterial community composition (Bray–Curtis abundance-weighted index) at week 0 differed among all treatments (PERMANOVA, Pseudo *F*_2,62_ = 8.1, *R*^2^ = 20.1%, *p* = 0.001; all pairwise *p* < 0.003), but not at week 4 (PERMANOVA all pairwise *p* > 0.12).

The Maryland Zoo in Baltimore provided 66 surplus-bred golden frogs, which we used to evaluate the ability for the engineered strain to colonize frog skin, produce violacein in this context and protect against Bd exposure. The golden frogs were divided into four treatment groups an uninfected control (control), Bd exposed (Bd), a probiotic treatment of the engineered core microbe *Diaphorobacter* 63F:vio after exposure to antibiotics/Bd exposed (Bd + 63F:vio), and a probiotic treatment including 63F:vio plus a natural consortium of seven anti-Bd golden frog bacteria (Fig. 3a & 1b). The golden frogs were maintained for 24 h after the last probiotic treatment prior to Bd exposure. To assess cutaneous microbial community dynamics, we swabbed each individual 1 day after probiotic treatment (immediately before Bd exposure) (week 0) and day 29 (week 4).

Bacterial species richness as measured by amplicon sequence variant (ASV) richness differed strongly among treatments at week 0 (Fig. 3b). As expected, individuals treated with 63F:vio after an antibiotic pre-treatment had lower ASV richness in their skin microbiome than the control and Bd + consortium golden frogs without an antibiotic pre-treatment. Four weeks after golden frogs were exposed to Bd, differences in bacterial species richness among all groups became less pronounced and were not statistically significant (Fig. 3b). Skin bacterial community composition among all treatment groups was similar at week 0, except for the antibiotic-treated group 63F:vio that differed (Fig. 3b, Fig. S1). At week 4, skin bacterial community composition was similar among treatments in abundance-weighted composition (Fig. 3b), but differed in presence/absence composition (Fig. S1), indicating that dominant taxa were similar in abundances among treatments, but that less abundant bacterial taxa were present in some treatment groups and absent in others.

**Fig. 4:**
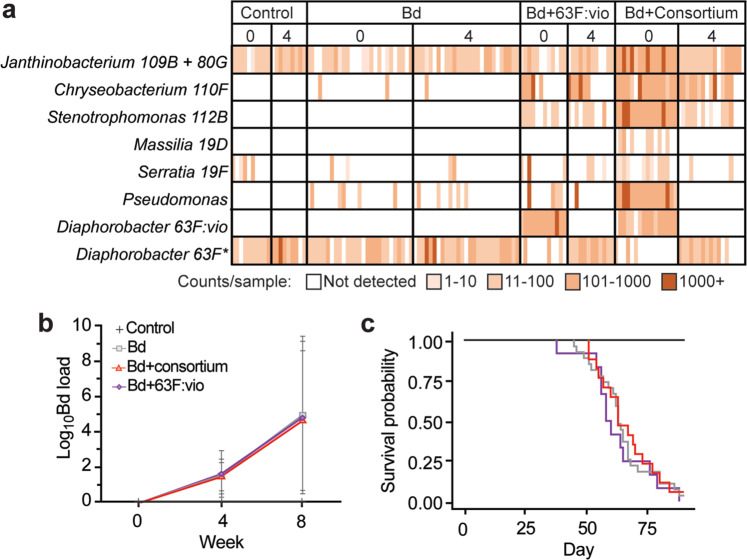
Probiotic, pathogen, and host outcomes. **a** Heatmap of probiotic species present on the frogs with different treatment regimes in weeks 0 and 4 respectively. Each stripe represents 16S frequency counts on an experimental animal (arranged in the same order for each timepoint). *Core *Diaphorobacter* refers to an ASV read present of on 86.3% of Week 0 and Week 4 individuals not treated with probiotics (**b**) Bd infection intensity in the 3 Bd-treatment groups and the uninfected control (Shown as log_10_ + 1-transformed zoospore equivalents ± standard error). Differences in Bd loads in Bd-exposed frogs between weeks 4 and 8 were significant (Repeated measures ANOVA, Chisq = 361.4, 1 DF = 1, *p* = <2e−16 ***, but the effects of probiotic treatment on Bd load in Bd-exposed frogs were not significant (Chisq = 0.903, 2 DF, *p* = 0.636). **c** Survival probability plot showing no difference in survival of *A. zeteki* between any of the Bd-exposed groups (Log-Rank test, Chisq = 0.6, 2 df, *p* = 0.7).

At the level of individual probiotics strains, the cocktail treatment approach resulted in *Chryseobacterium* 110F, and *Stenotrophomonas* 112B colonizing the skin well, and persisting until week 4 (Fig. 4a). *Janthinobacterium* was also present on control animals but increased in abundance in consortium-treated groups (mean count of 679 vs 84 in non-consortium groups, Kruskal–Wallis chi-squared = 33.5, df = 3, *p* = 2.471e-07). *Masilla* 19D and *Serratia* 19F did not colonize or persist on the frog skin (Fig. 4a). The *Pseudomonas* 103C and 63F:vio strains both colonized the skin at week 0, so were both present at the time of Bd inoculation, but did not persist to week 4 (Fig. 4a). The *Diaphorobacter* 63F:vio strain was distinguished from an unmodified, native *Diaphorobacter* strain by a single base-pair discrepancy in the 16S gene sequence allowing us to distinguish between them for analysis purposes.

We conducted a validation step to verify the presence and operation of the synthetic violacein pathway on golden frog skin. At week 0, we detected the pathway on 10/12 (83%) individuals only treated with the synthetic bacteria (Bd + 63F:vio), and 3/16 (18%) individuals treated with the probiotic consortium (Bd + consortium). No control animals (0/36) tested positive for the pathway. By week 4, we could not detect the synthetic violacein pathway, or the modified microbe on any golden frogs (Fig. 4a). The unmodified, native *Diaphorobacter* strain was well-represented on all individuals at the start of the experiment, but was extremely reduced by both probiotic treatments that included *Diaphorobacter* 63F:vio (Fig. 4a). By week 4 however, the *Diaphorobacter* 63F:vio had completely disappeared from the frogs’ skin, and was replaced by a pre-existing *Diaphorobacter* suggesting that there may have been an in vivo fitness cost to adding the violacein gene (Fig. 4a).

We also evaluated the effectiveness of the probiotic treatments to inhibit Bd growth and increase frog survival. All Bd-exposed animals became infected and remained infected for the duration of the experiment, with loads increasing over time and no differences among Bd-treatment groups (Fig. 4b). Similarly, there was no difference in survival between any of the Bd-treated groups, but all the control uninfected animals survived (Fig. 4c). We took a subset of skin swab samples from three individuals that had been treated with *Diaphorobacter* 63F:vio and 2 control individuals at week 0 and week 4 to test for violacein production via LC-MS. At both timepoints, we were unable to detect violacein in any samples. Thus, while the engineered bacteria were able to initially colonize the frog skin, they are lost rapidly and do not produce the desired compound in detectable quantities or have the desired therapeutic effect in this context.

## Discussion

We have made great progress in understanding microbiomes, and discovering associations likely connected with useful applied function. However, few studies have achieved success at manipulating microbiomes for a desired function, highlighting major gaps in our understanding of microbial community ecology and function [51,52]. Despite some successful, or partly successful examples of probiotic therapies being used to reduce Bd in amphibians [24,25,53,54] many probiotic treatments attempted so far in amphibians do not reduce Bd or improve survivorship when exposed to Bd [29,30,55–57]. There are many potential pitfalls when selecting a probiotic therapy requiring a species-specific approach [22], in particular, research on probiotic selection and persistence is required [58]. Major factors that affect probiotic colonization and persistence include host defenses, competition with resident microbes, and resource availability. In this study, we focused on developing and testing two novel strategies to overcome these key challenges that have affected the success of previous probiotic studies.

The probiotic treatments in this study temporarily altered the community composition of the frog skin microbiome, and the observed community-level changes in the synthetic *Diaphorobacter* 63F:vio treatment group are likely attributable to the antibiotic treatment which temporarily disrupted the pre-existing frog skin microbiome. Despite the apparently successful colonization of the *Diaphorobacter* 63F:vio and other seven probiotic strains at the time of Bd exposure, neither probiotic treatment limited Bd infection. It seems likely that none of these bacteria achieved a threshold density necessary to protective anti-Bd metabolite concentrations, a problem noted in many other probiotic therapies [12,29,59]. Our observations serve as a cautionary message to those attempting to develop probiotic therapies—it is difficult to predict in vivo microbiome functions derived from in vitro observations. Four out of eight probiotic microbes did not persist on the frog skin to week 4. This could be because the bacteria were poorly adapted to persist on the frog skin, despite initially being isolated from golden frogs, because of competition between probiotics or the pre-existing community [60], or that hosts upregulated skin defenses (e.g. antimicrobial peptides) to clear probiotic treatments [32].

In the case of the synthetic *Diaphorobacter* 63F:vio strain, the antibiotic treatment combined with direct supplementation of 63F:vio seems to have reduced the population of a prevalent pre-existing *Diaphorobacter*, but after week 4, the synthetic microbe had disappeared from the skin and been replaced by this pre-existing *Diaphorobacter*. We verified that the engineered and non-manipulated strains competed successfully in vitro. It is not clear if the genetic manipulation directly reduced the competitive ability of the microbe in vivo, but it is known that strains that have undergone many cycles of culturing can lose functional traits that affect adhesion [58]. In hindsight, further research into the competitive function and persistence of the synthetic microbe would have been informative prior to Bd exposure. However, the fact that there was no observed survival or reduction in infection rates, even though the probiotic treatment communities clearly included significant populations of the synthetic microbe at the time of Bd exposure make it doubtful that solving the persistence issue would have led to the desired disease outcome. The only core bacteria that we were successfully able to engineer (*Diaphorobacter* 63F), unfortunately, had a 1% average relative abundance in the 200 *A. zeteki* sampled prior to the experiment. It is worth considering that a better probiotic outcome may have been achieved by integrating the violacein gene pathway into a more abundant core bacteria (than *Diaphorobacter* 63F) or by using several engineered core microbes (Table 1).

The community-level microbiomes of consortium-treated golden frogs became less distinguishable with time after probiotic treatments. Prior to Bd exposure, the consortium treatments had a strong effect—being exposed to a probiotic consortium or the synthetic 63F:vio probiotic changed microbiome composition from the control state, but reverted to communities with common and numerically dominant bacteria similar to control animals later. Previous studies often detect changes in microbiome composition related to Bd [61,62], but in this case, we did not detect community-level microbiome differences due to Bd infection. Therefore, the post-Bd exposure homogenization of probiotically treated frog skin microbiomes are not likely due to Bd effects, but are simply reverting to pre-probiotic treatment states. We would normally expect to see an effect of Bd load on the microbial community [62], but the relative Bd loads at week 4 were comparatively low (Fig. 3a), so a lack of observed community-level effect is not remarkable.

Here, we implemented a strategy to introduce a genetic function needed by an endangered species into its microbiome. We used a consortium of probiotic candidates that were previously isolated from golden frogs, that were applied multiple times prior to Bd exposure, and for the first time, used a core skin microbe that had been successfully genetically modified to have a proven anti-Bd function. It is disappointing that we were unable to obtain the desired disease outcome. Indeed, it has been observed in other contexts, such as the human microbiome, that the establishment of an engineered bacterium in a persistent colonization of a host remains a challenge [63–65]. More basic research to understand the factors governing microbiome community assembly in relation to the host immune systems and pathogens will be needed to improve our ability to manipulate these systems in order to achieve a desired function. This example explores both the promise of microbial therapies and genetic engineering, and the challenges that we still face in understanding and manipulating microbiome communities and their function.

## Supplementary information

Supplementary Materials
