# Supplementary material for: Genetically modifying skin microbe to produce violacein and augmenting microbiome did not defend Panamanian golden frogs from disease
Source: ISME Commun. 2021 Oct 18;1:57. doi: 10.1038/s43705-021-00044-w (PMC9723765; doi:10.1038/s43705-021-00044-w)
Supplement: Supplementary file 1 — Supplementary Materials [file 43705_2021_44_MOESM1_ESM.pdf]

Supplementary Materials for

Genetically modifying skin microbe to produce violacein and augmenting microbiome did not defend Panamanian golden frogs from disease

Matthew H. Becker, Jennifer A. N. Brophy, Kevin Barrett, Ed Bronikowski, Matthew Evans, Emerson Glassey, Alyssa W. Kaganer, Blake Klocke, Elliott Lassiter, Adam J. Meyer, Carly R. Muletz-Wolz, Robert C. Fleischer, Christopher A. Voigt, and Brian Gratwicke

Table of Contents

Supplementary Tables

Supplementary Table 1: Sequences of plasmid used to construct *Diaphorobacter* 63F:vio 2
Supplementary Table 2: Probiotic bacteria isolated from Panamanian golden frog skin.......................... 3
Supplementary Table 3: Sequences of plasmid used to express GFP in *Diaphorobacter* 63F................ 6

Supplementary Figures

Figure S1: Bacterial community dynamics due to experimental treatment…………………..8

Supplementary Methods

Supplementary Methods……………………………………………………………………………………..9
Detection of violacein production in culture……………………………………………………………9
Detection of the violacein pathway on frog skin after probiotic treatments………………………...9
Bioinformatics and statistical analysis…………………………………………………………………10
Panamanian golden frog Bd exposure experiment husbandry protocol 13

Supplementary Table 1: Sequences of plasmid used to construct *Diaphorobacter* 63F:vio pJAB665

Sequence Name	DNA Sequence
5'homology 63F genome: met(CAG) tRNA	gtccggcgcagcgagaccttgcagtggctcttcctgcagacctcgagcatcgggcccatgctcggccagctctggtgg<br>ttccgccacggcacgagcacccgcaacgagcaggcgctggaacgctacacgcgggaggccaggcgcctgtacggcgtc<br>atcgagcggcgttttgccacccatccctacatcgcgggcgatgcctattcgatcgccgacatcgccatgttcccctgg<br>ttgcgcaccacggaggagctggggctggacgttgcggagtggccgcatgtgcaggcctggctgagcaaggtcggcgaa<br>cgccccgccgtgcagcgcgggctggcgctgctgcagccgcagatggcctgagccgctgtcctcgcttcacggaacccc<br>atttcccttctgaccccggcatcgctcatcgtgggcgacctggtccccgcattcccaggagattctcatggactggat<br>cctcttgcttctcgccggcctggtggaaatcggcatggccttcgccctcaaggccgccgccgggtggacccggccggt<br>gcctgccctgctgggcctgggggcggccattgtcagcatcgccctgctcacggcggcgctgcgcagcctgcccatcgg<br>cacggcctacgccatctggaccggcataggcgccatcggtgtcacggtcgtcggcatcatggtgttccaggaaagcgc<br>ctcgctgctgcgactggcctgcatcggaatgatctttgcagggatcattgggctgaagctgcaagggtgagtgccact<br>tcaagcggccgccttcgccttgaagaggcccggcgatgaaaaaagccacgcagtgcgtggctttttcgattggtggtc<br>gtagagggacttgaaccttcgacatcagcattatgaatgctgcgctctaaccaactgagctatacgaccctgttcatg<br>caacccacaggcttggaagcgtacttctgaaagtactgagggctggaatctggtggtcgtagagggacttgaaccttc<br>gacatcagcattatgaatgctgcgctctaaccaactgagctatacgaccggagacgaagattatagcccaggaatcga<br>gaac
-35 and -10 sites of promoter A1-LacO	cgcgcacatttccccgaaaagtgccacctgacgtctaagaaaccattattatcatgacattaacctataaaaataggc<br>gtatcacgaggccctttcgtcttcacctcgagaaaatttatcaaaaagagtgttgacttgtgagcggataacaatgat<br>acttagattcaattgtgagcggataacaatttcacacatctagaatagaggggt
vioD	atgaagattctggtcattggtgctggtccagctggtctggttttcgcatcccaactgaagcaggcacgccctttgtgg<br>gccattgacatcgtggagaagaatgacgagcaagaagtgctgggctggggtgtcgtgctgcctggccgtccgggtcag<br>cacccggcgaacccgctgtcctatctggatgcaccggagcgtctgaatccgcaatttctggaggacttcaaactggtg<br>catcataatgagccgtccttgatgtccacgggcgttttgttgtgcggcgtggagcgtcgcggtctggttcacgcgctg<br>cgcgataagtgccgcagccaaggcattgctattcgtttcgaaagcccgttgctggaacacggtgagctgccgctggcg<br>gactatgatctggtggtcctggctaatggtgttaatcacaaaaccgcgcatttcaccgaggctctggtcccgcaggtg<br>gactacggccgcaataagtacatttggtatggcactagccagctgttcgatcagatgaatctggtttttcgtacccat<br>ggtaaagatatctttatcgcgcatgcctataagtatagcgataccatgagcacgttcattgtcgaatgtagcgaagag<br>acttacgcacgcgcacgcctgggcgaaatgtccgaagaggcgagcgcagaatacgttgctaaggtgttccaggccgag<br>ctgggtggtcacggcctggtgagccagccgggtctgggttggcgtaacttcatgacgttgtctcatgaccgttgtcat<br>gatggtaagttggttctgctgggtgacgcgctgcaaagcggtcactttagcatcggccacggcaccacgatggccgtg<br>gtggtggcgcagctgctggttaaagcgctgtgtaccgaagatggtgtgcctgccgcgctgaaacgtttcgaagagcgt<br>gccctgccgctggtgcagttgttccgtggccacgcagacaacagccgcgtttggttcgaaaccgtcgaagagcgcatg<br>cacctgtcctcggcggaatttgtgcaaagcttcgacgcacgccgcaaaagcctgccgccgatgccggaagcactggcg<br>cagaatctgcgttatgctttgcagcgctgaaatgggtaacac
vioA	atgaaacattcttccgatatctgcattgttggtgctggtatttctggtttgacgtgcgcaagccatctgctggacagc<br>ccggcatgccgtggtctgagcctgcgtatctttgacatgcagcaagaagccggtggccgtatccgcagcaaaatgctg<br>gatggtaaggcaagcattgaactgggcgcaggtcgctactcccctcagttgcacccgcatttccaaagcgcaatgcag<br>cactatagccaaaagagcgaagtctatccgttcacccagttgaagttcaaatctcacgtgcagcaaaagctgaagcgc<br>gccatgaatgaactgtccccgcgtctgaaagagcatggtaaagagagctttttgcagtttgtcagccgttatcaaggt<br>cacgatagcgcggttggtatgatccgctctatgggttacgacgcactgttcctgccggatatcagcgcagaaatggcc<br>tacgacattgtgggtaagcacccggagatccagagcgtgacggacaacgacgcgaaccaatggtttgcagcggaaacg<br>ggctttgctggtctgattcagggcatcaaggctaaggttaaggcggcaggtgcgcgttttagcctgggttatcgtctg<br>ctgagcgtccgtaccgacggtgacggctacctgctgcaactggcaggtgacgacggctggaaactggagcaccgtacc<br>cgccatctgattctggcgattccgccgagcgcgatggcgggtttgaatgttgattttccagaagcctggtccggtgcg<br>cgctatggcagcctgccgctgtttaagggctttctgacgtacggtgagccgtggtggttggactacaaactggacgat<br>caggtgctgattgttgacaacccgctgcgcaaaatctatttcaaaggcgataagtacctgttcttctataccgatagc<br>gagatggcgaattactggcgcggttgtgtcgcggagggcgaggacggttacctggagcaaattcgcacccatttggct<br>agcgcactgggtatcgtccgtgaacgtatcccgcaaccgctggcacacgttcacaagtattgggcgcacggcgttgag<br>ttttgccgtgattctgatattgaccacccgagcgcactgtctcatcgcgacagcggtatcatcgcgtgctccgatgcg<br>tacacggagcattgtggttggatggagggcggtctgctgagcgcccgtgaggcaagccgtctgctgttgcagcgtatc<br>gccgcgtgagcttnnnnnn
vioB	atgagcattctggatttcccgcgtatccacttccgtggctgggcccgtgtcaatgcgccgaccgcgaaccgcgatccg<br>cacggccacatcgatatggccagcaataccgtggcgatggcgggtgagccgttcgacctggcacgccatcctacggag<br>ttccaccgtcacctgcgctccctgggtccgcgcttcggcttggatggtcgtgctgacccggaaggcccgttcagcctg<br>gccgagggctacaacgctgccggtaacaaccacttttcgtgggagagcgcaaccgttagccacgtgcaatggatggc<br>ggtgaggcggatcgtggtgacggtctggtcggtgctcgtttggcactgtggggtcactacaatgattatctgcgtacc<br>accttcaatcgtgctcgttgggtcgacagcgacccgacgcgccgtgacgctgcacaaatctatgcgggccaattcacc<br>attagcccggctggtgccggtccgggtacgccgtggctgtttacggcagacattgatgatagccatggtgcacgttgg<br>acgcgtggcggccacattgcagagcgtggcggccacttcttggatgaagagtttggtctggcacgcctgtttcagttc<br>tctgtgccgaaagatcacccacattttctgtttcacccgggtccgtttgattccgaggcctggcgtcgtctgcaattg<br>gctctggaggatgacgacgttctgggtctgaccgtgcaatatgcgttgttcaatatgagcaccccgcctcagccgaac<br>agcccggtttttcacgatatggtcggtgttgtcggtctgtggcgtcgtggtgaactggcgagctacccggctggtcgt<br>ctgctgcgtccgcgtcaaccgggtctgggtgacctgaccctgcgcgtcaacggtggtcgcgttgcgctgaatttggcg<br>tgtgccattccgttcagcactcgtgccgcgcagccaagcgcaccggaccgcctgaccccggacctgggtgccaaactg<br>ccgctgggcgatctgctgctgcgtgatgaggacggcgcactgttggcacgtgtgccgcaggctctgtaccaagactat<br>tggacgaatcacggtattgtggacctgccgctgctgcgcgaaccgcgtggtagcttgaccctgagcagcgaactggcg<br>gagtggcgtgagcaagactgggtcacccaaagcgacgcgtctaacctgtacctggaggcaccggatcgccgtcacggt

	cgctttttccctgagagcatcgcgctgcgcagctactttcgcggtgaagcgcgtgcgcgtccggatatcccgcatcgt<br>atcgagggcatgggcctggtcggcgtcgaatctcgtcaggatggcgacgctgcggaatggcgtctgacgggtctgcgt<br>ccgggtccggcacgcattgttctggacgatggtgccgaggcgatccctctgcgtgttctgcctgacgattgggcgctg<br>gatgacgcgaccgtcgaagaagtggattacgcctttttgtaccgccacgttatggcgtattacgagctggtgtatcca<br>ttcatgagcgacaaggtgttttccctggctgatcgttgcaaatgtgaaacgtacgcacgtctgatgtggcagatgtgt<br>gatccgcagaaccgcaacaagtcctattacatgccgagcacccgcgaactgtcggcaccgaaagctcgtttgttcttg<br>aagtatctggcccacgtggaaggccaggcacgcctgcaagcacctccgccagcgggtccggcacgcattgaatctaaa<br>gcccagttggcggcagagctgcgtaaagccgtcgacctggagctgtctgtgatgctgcaatacctgtacgcggcgtat<br>agcattccgaactatgcacagggccaacaacgtgttcgtgacggtgcgtggaccgccgagcagctgcaactggcgtgc<br>ggtagcggtgaccgtcgccgtgatggcggtattcgtgcagcactgctggaaattgctcatgaagaaatgattcattac<br>ctggtcgttaacaacctgctgatggccctgggcgagccgttctacgcgggtgtcccgctgatgggcgaagcggcacgt<br>caggcgtttggcctggacaccgagttcgctctggaaccgtttagcgaaagcacgctggcacgttttgttcgtctggaa<br>tggccgcactttatcccagcaccgggcaaatccatcgcggactgctatgccgccattcgtcaggcgtttttggatctg<br>ccggacttgtttggtggcgaggcaggtaagcgtggcggtgaacaccacctgttcctgaatgagctgaccaaccgtgcg<br>catccgggttatcaactggaagttttcgatcgcgactcggcgctgtttggtattgcatttgtgaccgatcagggcgaa<br>ggtggcgctctggacagcccgcactacgaacatagccattttcaacgtctgcgtgaaatgagcgcgcgtatcatggct<br>caaagcgcaccgttcgaaccggcgctgccggcgttgcgtaatccggttctggatgagagcccgggttgccaacgtgtc<br>gcagacggtcgtgcgcgtgcgctgatggcattgtaccaaggcgtttatgagctgatgtttgcgatgatggcgcagcac<br>ttcgccgtgaaaccgctgggtagcttgcgtcgcagccgcctgatgaacgcagcaatcgatctgatgaccggtctgttg<br>cgtccgctgagctgcgcgctgatgaacctgccaagcggcatcgccggtcgcacggccggtccgccgctgccgggtccg<br>gttgacacccgtagctatgacgactacgcgctgggctgtcgcatgctggcacgccgttgcgagcgtctgctggagcag<br>gcgagcatgctggaaccgggttggctgccggatgcgcagatggagctgctggatttctatcgtcgccaaatgctggac<br>ttggcgtgcggcaaactgagccgcgaggcctaagtaagatgat
vioC	atgaaacgtgcgattatcgttggtggcggcctggcgggtggcctgaccgcgatctacctggcgaagcgtggctacgaa<br>gtgcacgtcgtggagaagcgtggtgatcctctgcgcgatctgagctcttacgtggacgttgttagcagccgtgcgatc<br>ggcgtgagcatgaccgttcgtggtatcaagagcgttttggctgcgggcattccgcgtgcagagctggatgcgtgtggc<br>gaaccgatcgtggcaatggctttctccgtgggtggtcagtatcgcatgcgcgaactgaagccgttggaggatttccgt<br>ccgctgagcttgaaccgtgcggcgtttcaaaagctgctgaacaaatacgcgaacctggcaggcgttcgttactacttt<br>gagcataagtgcctggatgttgacctggatggtaagagcgtgttgattcagggcaaagatggtcagccgcagcgtctg<br>caaggtgacatgattatcggtgcggatggcgcccacagcgccgtccgtcaggcgatgcagagcggcctgcgtcgtttc<br>gagttccagcaaacgttcttccgccatggctacaaaaccctggttttgccggacgcgcaagcactgggttaccgtaaa<br>gacacgctgtactttttcggcatggattccggtggcctgttcgcgggtcgtgcggctacgatcccagatggtagcgtc<br>agcatcgccgtttgcctgccgtactcgggtagcccttccctgacgaccaccgacgaaccgacgatgcgtgcgttcttc<br>gatcgttacttcggtggcctgccgcgtgacgcgcgtgacgaaatgctgcgtcagtttctggcgaagccgagcaacgac<br>ctgattaacgtgcgctctagcacctttcactataagggtaatgtgctgttgctgggtgatgctgcgcatgcgactgcg<br>ccgttcctgggtcagggtatgaacatggcgctggaggacgcccgcacgtttgtcgagctgctggaccgccaccagggc<br>gaccaagacaaagcctttccggagttcacggagctgcgcaaagtccaggcagacgcaatgcaagacatggctcgcgcc<br>aactatgacgttttgagctgctcgaacccgatctttttcatgcgtgcgcgttacacgcgttacatgcattccaagttt<br>ccgggcctgtatccgccggatatggccgagaaactgtactttacgagcgagccgtacgatcgtctgcaacaaatccag<br>cgtaaacagaatgtttggtacaagattggtcgcgtgaattgagggcgtcgag
vioE	atggagaaccgtgagccaccactgttgccagcccgttggagcagcgcctatgtctcttattggagcccgatgctgccg<br>gatgaccagctgaccagcggctattgctggttcgactatgaacgtgacatctgtcgtattgacggcctgttcaatccg<br>tggagcgagcgtgatactggttatcgcctgtggatgtcggaggttggtaatgcggccagcggccgtacctggaaacaa<br>aaagtcgcctatggtcgtgagcgtaccgccctgggtgaacagctgtgtgagcgtccgctggatgatgagactggccct<br>tttgccgaattgttcctgccacgcgatgtcctgcgccgtctgggtgcccgtcacattggccgtcgcgtggttctgggt<br>cgcgaagcggacggttggcgttaccagcgcccaggtaaaggtccgagcaccctgtacctggatgcggcgagcggcact<br>ccactgcgcatggtcaccggcgatgaagcgtcgcgtgcaagcctgcgtgattttccgaatgtgagcgaggcggagatc<br>ccggacgcggttttcgcggccaagcgctaaaggt
Transcriptional terminator ECK120029600	ttcagccaaaaaacttaagaccgccggtcttgtccactaccttgcagtaatgcggtggacaggatcggcggttttctt<br>ttctcttctcaatcataggcaatacgatcgcatgtcctgcgtttctacaaactcttttgtttatttttctaaatacat<br>tcaaatatgcgcctgatgcggtattttctccttacgcatctgtgcggtatttcacaccgcatatcgacatccgccctc<br>accgccaggaacgcaaccgcagcctcatcacgccggcgcttcttggccgcgcgggattcaacccactcggccagctcg<br>tcggtgtagctctttggcatcgcctctcgcctgtcccctcagttcagtaatttcttgcatttgcctgtttccagtcgg<br>tagatattccacaaaacagcagggaagcagcg
Origin of transfer (oriT)	cttttccgctgcataaccctgcttcggggtcattatagcgatttttttcggtatatccatcctttttcgcacgatatac<br>aggattttgccaaagggttcgtgtagactttccttggtgtatccaacggcgtcagccgggcaggataggtgaagtagg<br>cccacccgcgagcgggtgttccttcttcactgtcccttattcgcacctggcggtgctcaacgggaatcctgctctgcg<br>aggctggccggctaccgccggcgtaacagatgagggcaagcggctcgagagattgccttgaatatatgacaatactga<br>taagataatgtatcttttatatagaagatatcgccgtatgtaaggatttcagggggcaaggcataggcagcgcgctta<br>tcaatatatctatagaatgggcaaagcataaaaacttgcatggactaatgcttgaaacccaggacaataaccttatag<br>cttgtaaattctatcataattgtggtttcaaaatcggctccgtcgatactatgttatacgccaactttcaaaacaact<br>ttgaaaaagctgttttctggtatttaaggttttagaatgcaaggaacagtgaattggagttcgtcttgttataattag<br>cttcttggggtatctttaaatactgtagaaaagaggaaggaaataataa
Kanamycin resistance gene aphA3	atggctaaaatgagaatatcaccggaattgaaaaaactgatcgaaaaataccgctgcgtaaaagatacggaaggaatg<br>tctcctgctaaggtatataagctggtgggagaaaatgaaaacctatatttaaaaatgacggacagccggtataaaggg<br>accacctatgatgtggaacgggaaaaggacatgatgctatggctggaaggaaagctgcctgttccaaaggtcctgcac<br>tttgaacggcatgatggctggagcaatctgctcatgagtgaggccgatggcgtcctttgctcggaagagtatgaagat<br>gaacaaagccctgaaaagattatcgagctgtatgcggagtgcatcaggctctttcactccatcgacatatcggattgt<br>ccctatacgaatagcttagacagccgcttagccgaattggattacttactgaataacgatctggccgatgtggattgc<br>gaaaactgggaagaagacactccatttaaagatccgcgcgagctgtatgatttttttaaagacggaaaagcccgaagag<br>gaacttgtctttcccacggcgacctgggagacagcaacatctttgtgaaagatggcaaagtaagtggctttattgat

	cttgggagaagcggcagggcggacaagtggtatgacattgccttctgcgtccggtcgatcagggaggatatcggggaa<br>gaacagtatgtcgagctatttttgacttactggggatcaagcctgattgggagaaaataaaatattatattttactg<br>gatgaattgttttagtacctagatttagatgtctaaaaagctttaactacaagctttttagacatctaatcttttctg<br>aagtacatccgcaactgtccatactctgatgttttatatcttttctaaaagttcgctagatagggggtcccgagcgcct<br>acgaggaatttgtatcgggcttccattcaggtcggggtggcccggctccatgcaccgcgacgcaacgcggggaggcag<br>acaaggtatagggcggcgaggcggctacagccgatagtctggaacagcgcacttacgggttgctgcgcaacccaagtg
3' homology 63F genome: enoyl CoA hydratase	acgaaaaatttcatggcggattgtgacggccgccatgagtttttcccggttggtgccggtcagacccggttagattcg<br>gtcaaaggggtcaggtgttggtgaagttggccttgcgcttgttcacgaaggcatccatgccttccttctggtcgttgg<br>tggcgaacagcccgtggaacaggcggcgctcgaacatcaggccgtcggagagcgagctctcgaaggcgcggttcacgg<br>tttccttggcggccatcacggcgacctgcgagaaatcgctgatctgcagcgccgcgcccagggtttcctccatgagct<br>tgtccagcggcacgacgcggctcacgaggcccgaacgctcggcctcggtggcgtccatcatgcggcccgtgagagcca<br>tgtccatggccttggacttgccgatcgcgcgcggcaggcgctgggtgccgccggcgcccggaatcacgcccagcttga<br>tttcaggctggccgaacttggcgttgtcggcggcaatgatgaagtcgcacatcatcgccagctcgcagccaccgccca<br>gcgcataacctgccacggcggcgatcacgggcttgcggatctggcggatggtttcccagttgcgggtgatgtagtcgt<br>tcttgtagacgtcggcaaagccgtacttggccatggccacgatgtcggcgcccgcggcaaatgccttttcgctgccgg<br>tcaggacgatgcagccgatcttctcgtccgcatcgaaggcgcgcagcgcatcgcccagctcgtccatgagctggtcgt<br>tcagcgcgttcagcgccttgggtcgattcagcgtgatgatgccgaccttctcggcttcggtgcgcacttcaatcattt<br>cgtaggccacggttatctca
Origin of replication R6K	gcagttcaacctgttgatagtacgtactaagctctcatgtttcacgtactaagctctcatgtttaacgtactaagctc<br>tcatgtttaacgaactaaaccctcatggctaacgtactaagctctcatggctaacgtactaagctctcatgtttcacg<br>tactaagctctcatgtttgaacaataaaattaatataaatcagcaacttaaatagcctctaaggttttaagttttata<br>agaaaaaaaagaatatataaggcttttaaagcttttaaggtttaacggttgtggacaacaagccagggatgtaacgca<br>ctgagaagcccttagagcctctcaaagcaattttgagtgacacaggaacacttaacggctgacatgggaattag

Supplementary Table 2: Bacteria isolated from Panamanian golden frog skin with anti-Bd properties that were selected for probiotic cocktail, and core skin bacteria. Isolates are lodged at the Agricultural Research Services Culture Collection (NRRL). 16S rRNA sequences are deposited in GenBank.

**Isolate ID**	**Role**	**Taxonomic Identification**	**% Bd Inhibition**	**NRRL #**	**GenBank Accession #**
19D	Probiotic	*Massilia violacea*	57.5%	B-65591	MT753025
19F	Probiotic	*Serratia marcescens*	100%	B-65592	MT753026
80G	Probiotic	*Janthinobacterium lividum* – purple	100%	B-65593	MT753027
109B	Probiotic	*Janthinobacterium lividum* - white	100%	B-65594	MT753028
110F	Probiotic	*Chryseobacterium sp.*	100%	B-65595	MT753029
112B	Probiotic	*Stenotrophomonas terrae*	96%	B-65596	MT753030
103C	Probiotic	*Pseudomonas soli*	100%	B-65597	MT753031
63F	Core microbe	*Diaphorobacter sp.*	51.1%	B-65598	MT753032
63F:vio	Synthetic core microbe & Probiotic	*Diaphorobacter sp.*	69.2%	B-65599	

Supplementary Table 3: Sequences of plasmid used to express GFP in *Diaphorobacter* 63F AJM_545

Sequence Name	DNA Sequence
lacI	tcactgcccgctttccagtcgggaaacctgtcgtgccagctgcattaatgaatcggccaacgcgcggggagaggcggt<br>ttgcgtattgggcgccagggtggtttttcttttcaccagtgagacgggcaacagctgattgcccttcaccgcctggcc<br>ctgagagagttgcagcaagcggtccacgctggtttgccccagcaggcgaaaatcctgtttgatggtggttaacggcgg<br>gatataacatgagctgtcttcggtatcgtcgtatcccactaccgagatatccgcaccaacgcgcagcccggactcggt<br>aatggcgcgcattgcgcccagcgccatctgatcgttggcaaccagcatcgcagtgggaacgatgccctcattcagcat<br>ttgcatggtttgttgaaaaccggacatggcactccagtcgccttcccgttccgctatcggctgaatttgattgcgagt<br>gagatatttatgccagccagccagacgcagacgcgccgagacagaacttaatgggcccgctaacagcgcgatttgctg<br>gtgacccaatgcgaccagatgctccacgcccagtcgcgtaccgtcttcatgggagaaaataatactgttgatgggtgt<br>ctggtcagagacatcaagaaataacgccggaacattagtgcaggcagcttccacagcaatggcatcctggtcatccag<br>cggatagttaatgatcagcccactgacgcgttgcgcgagaagattgtgcaccgccgctttacaggcttcgacgccgct<br>tcgttctaccatcgacaccaccacgctggcacccagttgatcggcgcgagatttaatcgccgcgacaatttgcgacgg<br>cgcgtgcagggccagactggaggtggcaacgccaatcagcaacgactgtttgcccgccagttgttgtgccacgcggtt<br>gggaatgtaattcagctccgccatcgccgcttccactttttcccgcgttttcgcagaaacgtggctggcctggttcac<br>cacgcgggaaacggtctgataagagacaccggcatactctgcgacatcgtataacgttactggtttcac
-35 and -10 sites of promoter A1-LacO	attcaccaccctgaattgactctcttccgggcgctatcatgccataccgcgaaaggttttgcaccattcgatggtgtc<br>aacgtaaatgcatgccgcttcgccttcgcgcgcgaattgcaggtaccatttatcagggttattgtctcatgagcggat<br>acatatttgaatgtatttagaaaaataaacaaataggggttccgcgcacatttccccgaaaagtgccacctgacgtct<br>aagaaaccattattatcatgacattaacctataaaaataggcgtatcacgaggccctttcgtcttcacctcgagaaaa<br>tttatcaaaaagagtgttgacttgtgagcggataacaatgatacttagattcaattgtgagcggataacaatttcaca<br>catctagaattaaagaggagaaattaacc
sfGFP	atgcgtaaaggcgaagagctgttcactggtgtcgtccctattctggtggaactggatggtgatgtcaacggtcataag<br>ttttccgtgcgtggcgagggtgaaggtgacgcaactaatggtaaactgacgctgaagttcatctgtactactggtaaa<br>ctgccggtaccttggccgactctggtaacgacgctgacttatggtgttcagtgctttgctcgttatccggaccatatg<br>aagcagcatgacttcttcaagtccgccatgccggaaggctatgtgcaggaacgcacgatttcctttaaggatgacggc<br>acgtacaaaacgcgtgcggaagtgaaatttgaaggcgataccctggtaaaccgcattgagctgaaaggcattgacttt<br>aaagaagacggcaatatcctgggccataagctggaatacaattttaacagccacaatgtttacatcaccgccgataaa<br>caaaaaaatggcattaaagcgaattttaaaattcgccacaacgtggaggatggcagcgtgcagctggctgatcactac<br>cagcaaaacactccaatcggtgatggtcctgttctgctgccagacaatcactatctgagcacgcaaagcgttctgtct<br>aaagatccgaacgagaaacgcgatcatatggttctgctggagttcgtaaccgcagcgggcatcacgcatggtatggat<br>gaactgtacaaatgaaacgcatgagaaagcccccggaagatcaccttccgggggctttttttattgcgctccttggccc<br>tccatccttagatag
Transcriptional terminator ECK120029600	ttcagccaaaaaacttaagaccgccggtcttgtccactaccttgcagtaatgcggtggacaggatcggcggttttctt<br>ttctcttctcaatcataggcaatacgatcgcatgtcctgcgtttctacaaactcttttgtttattttctaaatacat<br>tcaaatatgcgcctgatgcggtattttctccttacgcatctgtgcggtatttcacaccgcatatcgacatccgccctc<br>accgccaggaacgcaaccgcagcctcatcacgccggcgcttcttggccgcgcgggattcaacccactcggccagctcg<br>tcggtgtagctctttggcatcgcctctcgcctgtcccctcagttcagtaatttcttgcatttgcctgtttccagtcgg<br>tagatattccacaaaacagcagggaagcagcg
Origin of transfer (oriT)	cttttccgctgcataaccctgcttcggggtcattatagcgatttttttcggtatatccatcctttttcgcacgatatac<br>aggattttgccaaagggttcgtgtagactttccttggtgtatccaacggcgtcagccgggcaggataggtgaagtagg<br>cccacccgcgagcgggtgttccttcttcactgtcccttattcgcacctggcggtgctcaacgggaatcctgctctgcg<br>aggctggccggctaccgccggcgtaacagatgagggcaagcggctcgagagattgccttgaatatatgacaatactga<br>taagataatgtatcttttatatagaagatatcgccgtatgtaaggatttcagggggcaaggcataggcagcgcgctta<br>tcaatatatctatagaatgggcaaagcataaaaacttgcatggactaatgcttgaaacccaggacaataaccttatag<br>cttgtaaattctatcataattgtggtttcaaaatcggctccgtcgatactatgttatacgccaactttcaaaacaact<br>ttgaaaaagctgttttctggtatttaaggttttagaatgcaaggaacagtgaattggagttcgtcttgttataattag<br>cttcttggggtatctttaaatactgtagaaaagaggaaggaaataataa
Kanamycin resistance gene aphA3	atggctaaaatgagaatatcaccggaattgaaaaaactgatcgaaaaataccgctgcgtaaaagatacggaaggaatg<br>tctcctgctaaggtatataagctggtgggagaaaatgaaaacctatatttaaaaatgacggacagccggtataaaggg<br>accacctatgatgtggaacgggaaaaggacatgatgctatggctggaaggaaagctgcctgttccaaaggtcctgcac<br>tttgaacggcatgatggctggagcaatctgctcatgagtgaggccgatggcgtcctttgctcggaagagtatgaagat<br>gaacaaagccctgaaaagattatcgagctgtatgcggagtgcatcaggctctttcactccatcgacatatcggattgt<br>ccctatacgaatagcttagacagccgcttagccgaattggattacttactgaataacgatctggccgatgtggattgc<br>gaaaactgggaagaagacactccatttaaagatccgcgcgagctgtatgatttttttaaagacggaaaagcccgaagag<br>gaacttgtcttttcccacggcgacctgggagacagcaacatctttgtgaaagatggcaaagtaagtggctttattgat<br>cttgggagaagcggcagggcggacaagtggtatgacattgccttctgcgtccggtcgatcagggaggatatcggggaa<br>gaacagtatgtcgagctattttttgacttactggggatcaagcctgattgggagaaaataaaatattatattttactg<br>gatgaattgttttagtacctagatttagatgtctaaaagctttaactacaagctttttagacatctaatcttttctg<br>aagtacatccgcaactgtccatactctgatgttttatatcttttctaaaagttcgctagataggggtcccgagcgcct<br>acgaggaatttgtatcgggcttccattcaggtcggggtggcccggctccatgcaccgcgacgcaacgcggggaggcag<br>acaaggtatagggcggcgaggcggctacagccgatagtctggaacagcgcacttacgggttgctgcgcaacccaagtg
Origin of replication pBBR1	ctaccggcgcggcagcgtgacccgtgtcggcggctccaacggctcgccatcgtccagaaaacacggctcatcgggcat<br>cggcaggcgctgctgcccgcgccgttcccattcctccgtttcggtcaaggctggcaggtctggttccatgcccggaat<br>gccgggctggctgggcggctcctcgccggggccggtcggtagttgctgctcgcccggatacagggtcgggatgcggcg<br>caggtcgccatgccccaacagcgattcgtcctggtcgtcgtgatcaaccaccacggcggcactgaacaccgacaggcg<br>caactggtcgcggggctggccccacgccacgcggtcattgaccacgtaggccgacacggtgccggggccgttgagctt<br>cacgacggagatccagcgctcggccaccaagtccttgactgcgtattggaccgtccgcaaagaacgtccgatgagctt

	ggaaagtgtcttctggctgaccaccacggcgttctggtggcccatctgcgccacgaggtgatgcagcagcattgccgc<br>cgtgggtttcctcgcaataagcccggcccacgcctcatgcgctttgcgttccgtttgcacccagtgaccgggcttgtt<br>cttggcttgaatgccgatttctctggactgcgtggccatgcttatctccatgcggtagggtgccgcacggttgcggca<br>ccatgcgcaatcagctgcaacttttcggcagcgcgacaacaattatgcgttgcgtaaaagtggcagtcaattacagat<br>tttctttaacctacgcaatgagctattgcgggggggtgccgcaatgagctgttgcgtacccccctttttttaagttgttg<br>atttttaagtctttcgcatttcgccctatatctagttctttggtgcccaaagaagggcacccctgcggggttcccca<br>cgccttcggcgcggctccccctccggcaaaaagtggcccctccggggcttgttgatcgactgcgcggccttcggcctt<br>gcccaaggtggcgctgccccttggaaccccgcactcgccgccgtgaggctcgggggggcaggcgggcgggcttcgcc<br>ttcgactgcccccactcgcataggcttgggtcgttccaggcgcgtcaaggccaagccgctgcgcggtcgctgcgcgag<br>ccttgacccgccttccacttggtgtccaaccggcaagcgaagcgcgcaggccgcaggccggaggcttttccccagaga<br>aaattaaaaaaattgatggggcaaggccgcaggccgcgcagtcggagccggtgggtatgtggtcgaaggctgggtagc<br>cggtgggcaatccctgtggtcaagctcgtgggcaggcgcagcctgtccatcagcttgtccagcagggttgtccacggg<br>ccgagcgaagcgagccagccggtggccgctcgcggccatcgtccacatatccacgggctggcaagggagcgcagcgac<br>cgcgcagggcgaagcccggagagcaagcccgtagggcgggccgcgcgaattcgagctcggtaccgacgtagcccagcg<br>cgtcggccagcttgcaattcgcgctaacttacattaattgcgttgcgc

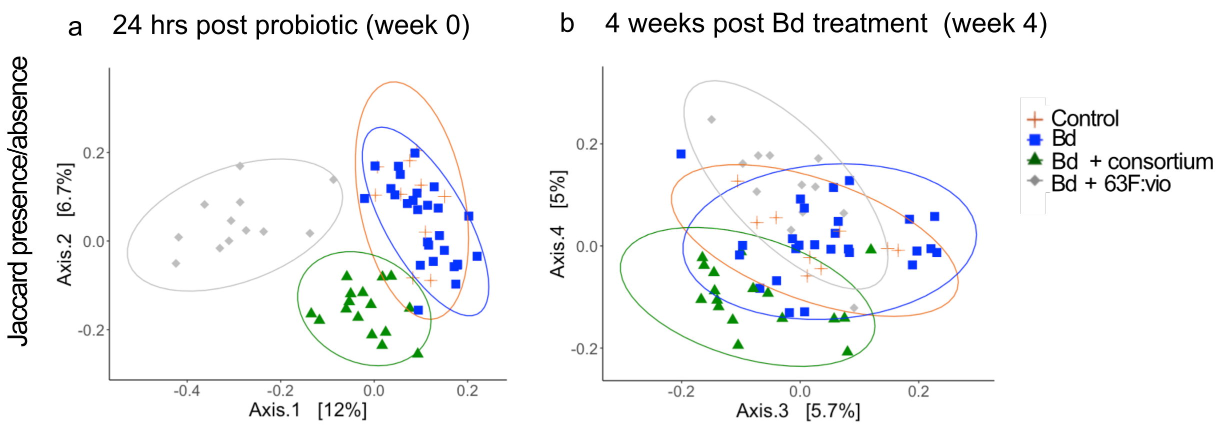

Figure S1: Bacterial community dynamics due to experimental treatment. Bacterial community composition (Jaccard presence-absence index) at week 0 (iii) differed among all treatments (Jaccard PERMANOVA, Pseudo $F_{2,62}$ = 5.7, R^2 = 15.6%, p = 0.001, all pairwise adj. p < 0.003), and at week 4 (iv) differed among most treatments at week 4 (iv) (Jaccard PERMANOVA, Pseudo $F_{3,60}$ = 2.1, R^2 = 9.4%, p = 0.001, all pairwise adj. p < 0.006: except Bd+ compared to control).

Supplementary methods

Detection of violacein production in culture.

Single colonies were inoculated into 50 mL of LB medium in 250 mL culture flasks and incubated ~42 h at 30°C and 250 rpm. Kanamycin was added to the culture flasks when growing *Diaphorobacter* containing a plasmid-borne violacein pathway. Cells were harvested by centrifugation and resuspended in 500 μL methanol to lyse cells and extract violacein. Cell lysate was then centrifuged for 10 minutes at 47000 r.p.m. Supernatant was then transferred to a fresh 1.5 mL Eppendorf tube and spun for 5 minutes at 15000 r.p.m. to remove any additional cell debris. Samples were analyzed via liquid chromatography coupled to mass spectrometry (LC-MS) using a 1260 Binary Pump (Agilent, Santa Clara, CA, USA) in low dwell volume mode, an Agilent column oven heated to 40 °C, and a 6420 Triple Quadrupole Mass Spectrometer with an ESI source (Agilent, Santa Clara, CA, USA). 10 μl of sample was injected onto a 2.6 μm particle size, 50 x 2.1 mm i.d., Kinetic Core-Shell Evo-C18 column (Phenomenex, Torrance, CA, USA) and analyzed using the following method: 0.0-0.5 min at 25% (vol/vol) acetonitrile, 0.5-6.5 min at 25 to 85% (vol/vol) acetonitrile gradient, 6.5-7.0 min at 85 to 95% (vol/vol) acetonitrile gradient, 7.0-8.0 min at 95% (vol/vol) acetonitrile, with 1.0 min of re-equilibration. Flow rate was maintained at 0.5 mL/min. Mobile phases were (1) acetonitrile with 0.1% (vol/vol) formic acid and 0.1% (vol/vol) H2O and (2) H2O with 0.1% (vol/vol) formic acid. The ESI source was run with a desolvation temperature of 350 °C, a drying gas flow rate of 12 L/minute, a nebulizer pressure of 15 psi, and a capillary voltage of 4,000 V. The MS was run in MRM mode using Unit resolution for both precursor and product ion quadrupoles, positive ionization mode, and cell accelerator voltage of 4. Compound specific parameters were optimized using the Agilent Optimizer software and are as follows (mass-to-charge (m/z) listed in Daltons per electron volt): violacein (quantifier ion - used to integrate peak): 344.1 (precursor ion) m/z > 301.1 (product ion) m/z, CE (collision energy in electron volts) 25 eV, FV (fragmentation voltage) 150 V; violacein (qualifier ion - used to validate/qualify quantifier ion): 344.1 m/z > 316.2, CE 25 eV, FV 150 V; deoxyviolacein (quantifier ion): 328.1 m/z > 285.0 m/z, CE 29 eV, FV 123 V; deoxyviolacein (qualifier ion): 328.1 m/z > 300.1 m/z, CE 25 eV, FV 123 V. Post-acquisition data processing was done using MassHunter Quantitiative Analysis software (Agilent, Santa Clara, CA, USA) to extract MRM chromatograms and integrate peaks. No peaks were observed for samples (data not shown).

Detection of the violacein pathway on frog skin after probiotic treatments.

We screened isolated DNA from individuals at weeks 0 and 4 for presence of the violacein pathway. PCR reactions were done at 20 or 30 μL scale, with 1X master mix, 1 μM of the primer soJBRO169 (cagctgttcgatcagatgaatctggtttttc) -soJBRO160 (ctttaccatgctctttcagacgcgggg), and 2 μL of isolated DNA. A touchdown PCR was run, with 7 cycles between 72 °C and 65 °C (-1 °C per cycle) and 28 additional cycles at 65 °C. We verified that the PCR products were the correct sequence by extracting and purifying 3 PCR bands (the positive control and two additional samples) and sequencing the extracted DNA.

We selected a subsample of 3 Bd + 63F:vio treatment individuals with high Diaphorobacter 63F:vio reads, 1 control animal with high *J. lividium* reads, and 1 control animal with no *J. lividium* detected to screen for violacein. We extracted skin swabs (Bd + 63F:vio individuals) or skin tissue (control individuals) with 30-50 μl of MeOH in order to retrieve 30ul for analysis. All samples were centrifuged (21,130 g, 5 min), and then transferred to a 96-well plate for analysis.

Bioinformatics and statistical analysis.

To analyze the culture-independent data, we used QIIME 2.0 (1) to process the reads and R version 3.5.3 for subsequent analyses. All raw fastq sequence files were deposited in the Sequence Read Archive (PRJNA698905). In QIIME, we demultiplexed reads and assigned reads to samples based on unique indices (2) and filtered out low quality reads using dada2 (3). Sequences were categorized into amplicon sequence variants (ASVs) using dada2 and taxonomy was assigned by aligning ASVs with the SILVA database and then filtering out mitochondria and chloroplasts (4). A phylogenetic tree was built using the fasttree algorithm (5). Files created in QIIME were exported and then imported into R, where all subsequent analyses were conducted. We used the 'decontam' package to remove potential contaminants using the frequency-based method; four ASVs were identified as potential contaminants and removed from subsequent analyses.

We assessed differences in alpha diversity and beta diversity among treatments at two time-points: week 0 and week 4 (Figure 3b). At week 4, we had four treatments: Bd (n = 26), controls (n = 9), Bd + consortium (n = 17), and Bd + 63F:vio (n = 12). We quantified alpha diversity using bacterial ASV richness. We used ANOVAs (all data met assumptions) to determine the effects of treatment on ASV richness, and conducted post hoc analyses using the Tukey's 'Honest Significant Difference' (HSD) method. We quantified community composition (beta diversity) using Jaccard and Bray-Curtis indices, and used a PERMANOVA to determine the effects of treatment on community composition using 'adonis' function in vegan package (6) and post hoc analyses using 'pairwise.adonis' function in pairwise Adonis package (7). Prior to conducting Bray-Curtis analyses, we performed proportion normalization on raw sequence counts to correct for biases associated with unequal sequencing depth on this abundance-weighted metric (8, 9). Variation was minimal (7.6x difference in sequencing depth), so all other alpha and beta diversity metrics should be minimally impacted by sequence coverage (8, 9).

We used a custom BLAST in Geneious 10.2.2 to detect the probiotic bacteria that occurred on the skin at Week 0 and Week 4 (Figure 5a). We matched 16S rRNA gene sequences of the probiotic bacteria (Sanger sequencing from pure cultures) to the 16S rRNA gene sequences of the skin microbiome (Illumina high-throughput sequencing from DNA extracts of skin swabs). We used a megablast program, having Geneious return results as query-centered alignment data only and returning only the top hit. Microbiome-

derived sequences that matched at 100% similarity were considered the same taxon as the probiotic-applied bacteria.

To generate a phylogenetic tree of the isolates cultured from golden frog skin (Figure 1a), we generated an alignment of the cultured isolates' 16S rRNA gene sequences with an Archaeal outgroup sequence (*Methanocaldococcus jannaschii*) using Ribosomal Database Project (RDP). We cleaned up the RPD alignment to remove special characters using a Perl script (10). We then used this clean alignment file to build a 16S rRNA gene tree with FastTree 2.1.11 in Geneious Prime 2019.2.3 using default parameters. The tree was rooted with the Archaeal outgroup sequence. We visualized and annotated the tree using Interactive Tree of Life (11).

To assess differences in Bd infection intensity due to treatment, we $\log_{10}$+1 transformed Bd loads of frogs that survived until week 8 and used a repeated measures ANOVA analysis in the 'nlme' and 'car' packages in R (12) (Figure 5b). To assess differences in survivorship between the Bd-exposed groups, we used survdiff in the R package survival for R (13) to conduct a log-rank test (14) (Figure 5b).

References

1. E. Bolyen, *et al.*, Reproducible, interactive, scalable and extensible microbiome data science using QIIME 2. *Nat. Biotechnol.* **37**, 852–857 (2019).
2. J. G. Caporaso, *et al.*, Global patterns of 16S rRNA diversity at a depth of millions of sequences per sample. *Proc. Natl. Acad. Sci. U. S. A.* **108**, 4516–4522 (2011).
3. B. J. Callahan, *et al.*, DADA2: High-resolution sample inference from Illumina amplicon data. *Nat. Methods* **13**, 581–583 (2016).
4. Q. Christian, *et al.*, The SILVA ribosomal RNA gene database project: improved data processing and web-based tools. *Nucleic Acids Res.* **41**, D590-D596. (2013).
5. M. N. Price, P. S. Dehal, A. P. Arkin, FastTree 2 - Approximately maximum-likelihood trees for large alignments. *PLoS One* **5** (2010).
6. J. F. Oksanen, *et al.*, vegan: Community Ecology Package. *R Packag. version 2.5-6*, https://CRAN.R-project.org/package=vegan (2019).
7. P. M. Arbizu, pairwiseAdonis: Pairwise Multilevel Comparison using Adonis. *R Packag. version 0.0.1.* (2017).
8. S. Weiss, *et al.*, Normalization and microbial differential abundance strategies depend upon data characteristics. *Microbiome* **5**, 27 (2017).
9. P. J. McMurdie, S. Holmes, Waste Not, Want Not: Why Rarefying Microbiome Data Is Inadmissible. *PLoS Comput. Biol.* **10**, e1003531 (2014).
10. M. I. Dunitz, *et al.*, Swabs to genomes: A comprehensive workflow. *PeerJ* **2015**, e960 (2015).

11. I. Letunic, P. Bork, Interactive Tree of Life (iTOL) v4: Recent updates and new developments. *Nucleic Acids Res.* **47**, W256–W259 (2019).
12. J. Fox, S. Weisenberg, Package “car,” 2019. Description Functions to Accompany J. Fox and S. Weisberg, An R Companion to Applied Regression, Third Edition, Sage, Ed. (2019).
13. T. M. Therneau, T. Lumley, R: Survival Analysis 3.1-8. *Surviv. Anal.*, https://github.com/therneau/survival (2019).
14. D. P. Harrington, T. R. Fleming, A Class of Rank Test Procedures for Censored Survival Data. *Biometrika* **69**, 553 (1982).

Supplementary Methods: Panamanian golden frog Bd exposure experiment husbandry protocol

Species: Panamanian Golden Frog (*Atelopus zeteki*) Accession No: #307225.

Description: 60-80 subadult-adult frogs will be housed in Experimental Animal Holding Rooms # 2 & 3. Frogs will be individually housed in plastic enclosures (Alternative Design, large plastic mouse cages measuring 29.2 cm x 19 cm x 12.7 cm with low-profile, filter-top lids https://www.altdesign.com/product-category/rodent-ivc/plastic-large-mouse-cages/). Each container will include a damp paper towel, reverse osmosis (RO) water reconstituted with minerals and salts, and a small hide (opaque plastic flower pots measuring 5.7 cm x 5.7 cm x 8.3 cm). Containers will be angled using a wooden dowel at the back end of the tank to create wet and dry areas in the enclosure. Enclosures will be positioned on metroracks and numbered. Disease treated animals will be maintained on racks separately from untreated frogs. Every shelf on the racks will be fitted with 4ft ZooMed UVB 10 bulbs placed on a 12-hour automated lighting cycle from 6AM-4PM.

We will use a reverse osmosis (RO) unit AquaFX Dolphin RO System 300 gpd for deionizing water in Experimental Animal Room # 2. The entire building is connected to a central HVAC system with a backup generator. There will also be three Hobo humidity and temperature meters recording max, min and current humidity and temperature in the room and in at least one cage. Temperatures may range between 20-24 ˚C with a target of 22˚C. A portable air conditioner will be used to ensure that the room remains within this temperature range. Humidity within the enclosure/tanks should be 70% or higher. There will be one small, regularly cleaned work table and a chair in the room so that no items are placed on the floor while working cages.

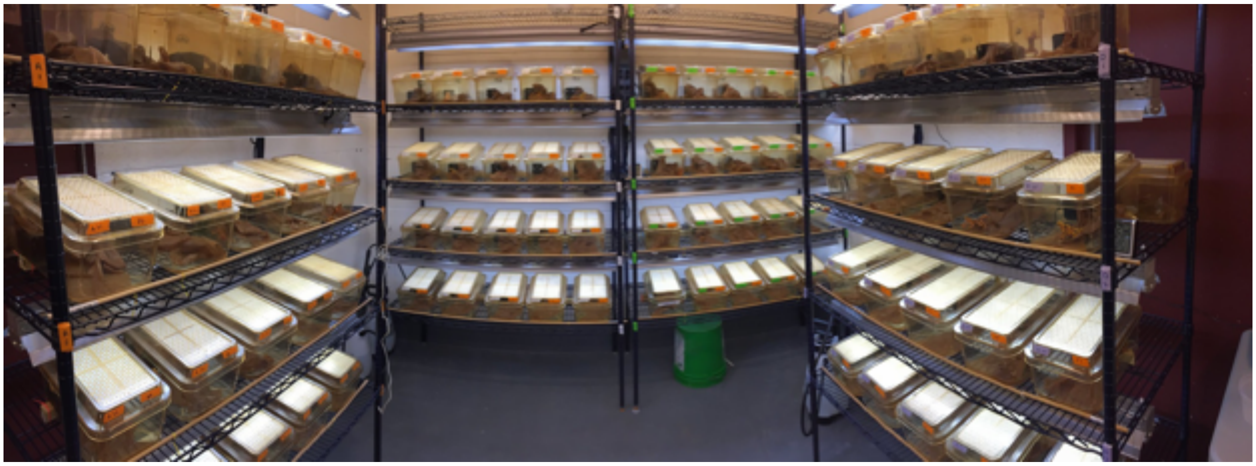

Room #3 setup with golden frog cages.

The room and all equipment entering the room will be cleaned with 1 part bleach: 9 parts water before establishment of the research colony.

Water Quality: Water is filtered through an RO unit and placed into a 90 liter reservoir. Once the reservoir is full it will be reconstituted using the following formula:

Recipe specific to the 90-liter reservoir
3.56 Calcium chloride
4.19 g Magnesium sulfate
3.23 g Potassium bicarbonate
2.69 g Sodium bicarbonate

Once a reservoir has been emptied it should be refilled while the other reservoir is being used, so that there is always an abundant supply of clean, reconstituted water on hand. Refilling takes several hours and can be accomplished overnight. Water is supplied to the cages using a 90L trash can on wheels and a dispensing jug. Water will initially be tested for chlorine, pH, ammonia, nitrates and nitrites using a Hach colorimeter in the Reptile Discovery Center. Subsequently, each batch of water will be tested for chlorine using Hach chlorine test strips and a conductivity meter prior to mixing each batch. If any chlorine is detected in the RO water or conductivity rises above baseline levels, water will be subject to further testing before being used on frogs.

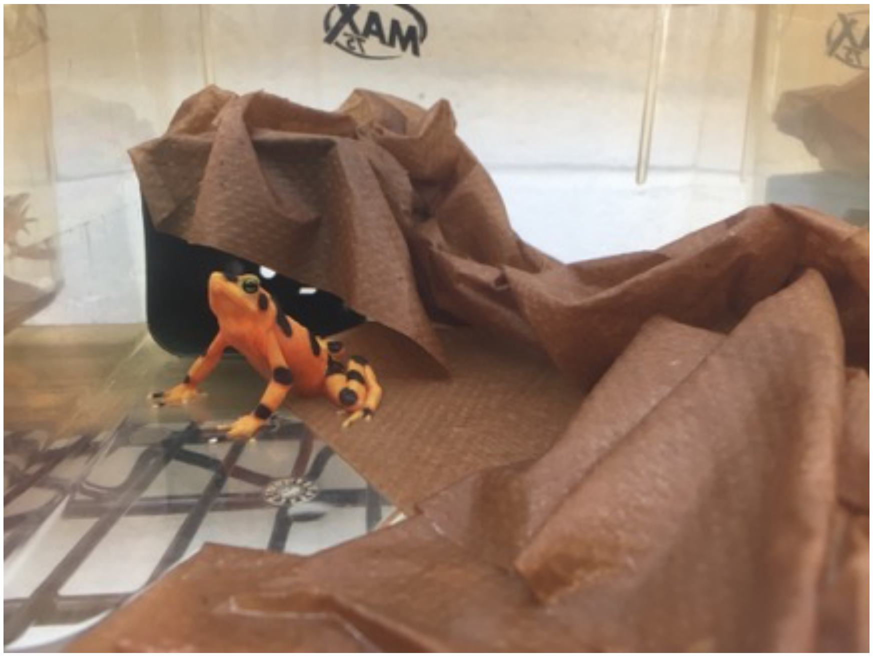

Golden frog in mouse cage with hide and moistened paper towel.

Cautions/Warnings/Dangers: The frogs are adept climbers and bin lids should be fitted tightly to prevent escapes. The floor drain in Experimental Animal Room # 3 will be covered with fine mesh to prevent the loss of any escapees down the drain. Ensuring good water quality for the frogs is essential for this experiment; RO water strips most of the dissolved salts from the water, and therefore increases the osmotic potential and could be harmful to the frogs if not re-constituted using dissolved salts. A bleach solution of at least 1 part bleach to 9 parts water is advocated for cleaning and sterilizing containers and equipment, but it is highly toxic to amphibians; all equipment treated with bleach should be thoroughly rinsed, and dried. Amquel Plus (Sodium thiosulphate active ingredient) will be added 5 ml per 10 gallons of rinsing water to dechlorinate any residual bleach.

Maintenance of proper temperature and humidity levels is essential to both the health of the frogs and the procedure of the trials, so doors should not be left open for prolonged periods of time. Temperatures will be monitored daily to assure that they remain between established high/low set-points. Temperature alarms linked to the Boiler Plant are set at 22°C. Should there be any variance beyond the set-points the staff will have on hand a phone-tree with contact information for OFMR/HVAC (on duty 24/7 at the zoo's Boiler Plant) as well as all husbandry staff (RDC Curator, RDC Biologist, Senior Curator, SCBI Building Manager, PI Scientist, co-PI Scientist, RDC Keeper).

Medical/Health Issue Alerts: Panamanian golden frogs are prone to suffer from tetany (they tend to drag their hind legs and loose mobility). It is unclear precisely what causes this, but treatment includes calcium supplements and exposure to UV light. If any individuals develop tetany, these individuals will be monitored closely. Any dead frogs should be removed immediately from the container, swabbed, and preserved in formalin. Any health cases of tetany, or any other unexpected symptoms will be reported to the RDC curator and biologist.
All animals that die will be swabbed to sample cutaneous microbial communities, metabolites, and host RNA. Carcasses will be preserved for full histopathology analysis and measurements. All surviving animals with high infection intensities and frogs that are moribund will be euthanized with saturated buffered MS-222 for histopathological comparisons. Euthanasia will be performed by a veterinarian using an intracoelomic overdose of MS-222. All carcasses will be preserved in 10% formalin and accessioned to the pathology collection as a single batch at the termination of the experiment.

Feed Storage: Crickets are housed in 2 polycarbonate bins in the Experimental Animal Room # 2. Water is provided from a bottle and sponge, crickets are provided with cricket diet for gut loading. New crickets are ordered weekly. Wingless fruit flies should be kept in the same conditions as the crickets, gut loading not necessary.

The purpose of housing and feeding insects prior to the inclusion in diets is to compensate for any nutrients that are lacking in the natural composition of those insects. Injecting, dusting, and gut loading are all ways to add nutritive values to insects. Although some of these methods have shortcomings (injecting is very time intensive, dusting is relatively temporary and unreliable), gut loading allows the insects to incorporate additional nutrients through natural feeding behavior.

Free-ranging insectivorous animals consume a wide variety of insect prey in the wild, and this variety allows for adequate nutrients in their diets. In captivity, only a few species of insects are available and few, if any, provide adequate nutrients to captive insectivores. By gut loading, we can improve the nutrient content of those few insects that we have available in order to provide the best nutrient package possible to the animal. The insect meal is not designed to be nutritionally complete for the insect (Bernard and Allen 1997), nor does it appear to be particularly palatable. For this reason, to ensure optimal gut loading, following guidelines established to insure adequate insect meal consumption is imperative. We will follow the NZP cricket and mealworm gut loading protocol.

All animals will be fed every other day.

1. The temperature for the areas in which are held should remain relatively constant between 80-87 degrees F in order to insure optimal gut loading (Bernard and Allen, 1997). This temperature is not necessarily recommended to promote the longevity of the insects (as their days are already numbered, anyway), but to insure adequate gut loading. A dark environment is preferred.
2. Crickets should be maintained with access to the appropriate supplement (insect meal / cricket diet) for between 48-72 hours in order to achieve optimal gut loading (Ca:P ratio between 1:1 - 2:1, 0.88+% Ca, 0.88+% P). Allen and Oftedal (1989) reported that the Ca:P ratio in unsupplemented crickets ranged from 0.06:1 to 0.13:1, whereas the goal is 1.5:1 to 2:1 (Bernard and Allen 1997, Allen 1992, Trusk and Crissey, unpublished).
3. Produce should be eliminated from the holding bins. Water should not be offered in the form of produce because the crickets would preferentially consume the produce item over the insect meal. If space in the GI tract is occupied by produce material (lesser nutritive value), less room is available for insect meal (greater nutritive value). Water can be offered in a chick feeder covered with a screen or filled with rocks (to prevent drowning), or a bottle with a sponge inserted in its mouth. This will encourage the insects to consume the meal rather than the fruit, and provide them with an adequate source of water (enabling optimal consumption of the meal).
4. Supplemental food sources (i.e sweet potatoes, oranges, etc) are not recommended for the same reasons that produce is not recommended as a moisture source.

Feeding Instructions: Feeding of fruit flies, pinhead or 10-day old crickets are to be fed ad-lib every other day to each tank (10-20 insects depending on size). Food should be offered on an alternated

schedule. Most food should be offered in sufficient quantities that it is always available to the animals for consumption. In this way frogs have a continuous availability of food, with no need to correct food allocations for body size. Any observed morbidity should be a result of the experimental treatment rather than lack of food.

Security and Biosecurity: All gates, latches, lids locks and doors must be checked and monitored for CLAWS (closed, locked, access, water and safety [both animals and staff]) to ensure containment and human and animal safety. The DCB building is not accessible by the public and only authorized personnel can enter the building. Additionally, the facilities in the basement have controlled-access and only authorized personnel within the building will have access to these rooms. The rooms will be kept locked at all times.
PPE - Dedicated foot-ware, gloves and scrubs will be provided for routine husbandry inside Experimental Animal Room # 2, if dedicated foot ware is not used, 10% bleach footbaths should be used. Frogs will only be handled with powder-free nitrile gloves, rinsed in re-constituted RO water, or with 70% ethanol and allowed to dry. No other amphibians should be handled or serviced by RDC staff the same day after entering study rooms.

Biohazardous Waste:

1. Each time the room is cleaned (every other day), the day's waste will be placed in an appropriately sized smaller plastic bag, the bag will be sealed, and then placed into the larger biohazard box.
2. The red bag within the biohazard box should also be tied daily as secondary insurance that the crickets cannot escape.
3. The biohazard box will be placed within Experimental Animal Room # 2.
4. When the weight of the box is nearing 50lbs, the red biohazard bag should be closed with a single knot tied to itself and the box must be taped closed (as instructed on the box).
5. These boxes will be stored in Experimental Animal Room # 2 until scheduled pickup (to be scheduled with IPM).

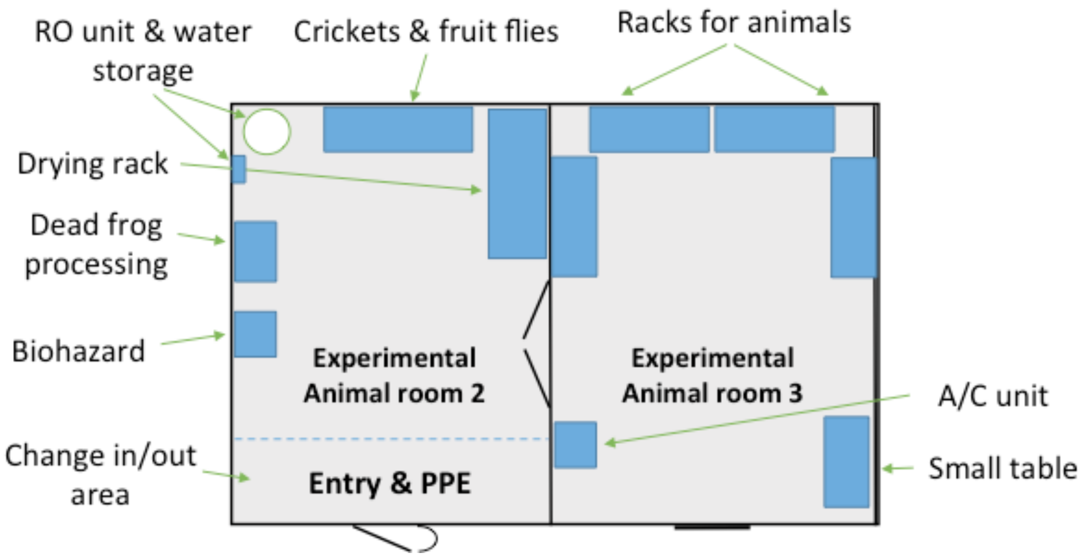

Experimental room layout

Daily Routine: *Work cages starting with untreated individuals and ending with frogs exposed to probiotic and pathogen*

Daily: Mist each cage heavily once using reconstituted RO water from a reservoir and a hand mister. Take care not to touch inside of the tanks and as a precaution, disinfect misting nozzle with 70% alcohol solution when switching between experimental treatments. Wear gloves at all times and change gloves between experimental treatments.

Mon – Wed – Fri: Feed frogs approximately ¼ tsp crickets or fruit flies. Replace brown paper towels and water in each cage. If frog leaps onto floor or other non-sterile surface, spray the frog down with clean water using mister, then replace the frog into its container. Spray all surfaces touched by the frog with 70% ethanol. When waste bucket is filling up, 1-part bleach should be added for every 9 parts water in this waste. The mixture, including paper towels, should be mixed and then the paper towels should be removed squeezed to remove excess water then be placed in a disposal bag, along with all gloves and other dry waste. This waste should be sealed in the bag with a knot to prevent any escaping crickets then placed in the biohazard box stored in Experimental Animal Room # 2. The bleached water may be disposed of down the drain and should not generally be added to the biohazard bags which leak with excessive amounts of liquid inside them. Remember that minimal handling is desirable to reduce stress to frogs. In addition to cleaning and feeding times note any unusual frog behavior in logbook.

Changing cages: Every 14 days containers will be switched out and replaced with fresh containers. Dirty cages, lids, and hides will be scrubbed in a warm solution of 9 gallons water: 1-gallon bleach, then rinsed in 20 gallons of fresh RO water treated with water conditioner to neutralize any chlorine. The cages and

hides will then be dried on drying racks inside Experimental Animal Room # 2 where frogs will not be housed. Sterile cages and hides should be handled only with gloves, cleaned with 70% alcohol if any non-sterile surfaces have been touched. New cages will be labeled appropriately, and care taken to handle them only with gloves.

Observations required: Daily records of min/max temperature and humidity readings are recorded for the room and inside a polycarbonate cage. All records will be saved for reference. Temperatures may range between 20-24 ˚C with a target of 22˚C. Humidity within the enclosure/tanks should be 70% or higher. Record max/ min and current temp and humidity for room and polycarbonate cage in logbook. Adequate temperature and humidity will be maintained a week prior to receiving animals. Target humidity will be maintained with placing wet unbleached paper towels in animal enclosures and misting.

C**leaning of room:** The floor of the room should similarly be cleaned with 1-part pleach:9 parts water and rinsed using clean tap water.
